# Activated protein C reverses epigenetically sustained p66^Shc^ expression in plaque-associated macrophages in diabetes

**DOI:** 10.1038/s42003-018-0108-5

**Published:** 2018-08-06

**Authors:** Khurrum Shahzad, Ihsan Gadi, Sumra Nazir, Moh’d Mohanad Al-Dabet, Shrey Kohli, Fabian Bock, Lukas Breitenstein, Satish Ranjan, Tina Fuchs, Zuhir Halloul, Peter. P. Nawroth, Pier Giuseppe Pelicci, Ruediger C. Braun-Dullaeus, Eric Camerer, Charles T. Esmon, Berend Isermann

**Affiliations:** 1 Institute of Clinical Chemistry and Pathobiochemistry, Otto-von-Guericke-University, Leipziger Straße 44, 39120 Magdeburg, Germany; 2 Department of Biotechnology, University of Sargodha, Sargodha, 40100 Pakistan; 3 Department of Medicine, Vanderbilt University Medical Center, 37232 Nashville, TN USA; 4 Institute for Clinical Chemistry, University of Heidelberg Medical Faculty Mannheim, 68167 Mannheim, Germany; 5 Division of Vascular Surgery, Department of General, Abdominal and Vascular Surgery Otto-von-Guericke-University, Leipziger Straße 44, 39120 Magdeburg, Germany; 6 Department of Internal Medicine I and Clinical Chemistry, German Diabetes Center (DZD), University of Heidelberg, 69120 Heidelberg, Germany; 7 Department of Experimental Oncology, European Institute of Oncology, Via Ripamonti, 435, 20141 Milan, Italy; 8 Department of Internal Medicine, Division of Cardiology and Angiology, Otto-von-Guericke-University, Leipziger Straße 44, 39120 Magdeburg, Germany; 9 INSERM U970, Paris Cardiovascular Research Centre, 75015 Paris, France; 10 Coagulation Biology Laboratory, Oklahoma Medical Research Foundation, and Department of Pathology and Department of Biochemistry & Molecular Biology, University of Oklahoma Health Sciences Center, Oklahoma City, 73104 OK USA

## Abstract

Impaired activated protein C (aPC) generation is associated with atherosclerosis and diabetes mellitus. Diabetes-associated atherosclerosis is characterized by the hyperglycaemic memory, e.g., failure of disease improvement despite attenuation of hyperglycaemia. Therapies reversing the hyperglycaemic memory are lacking. Here we demonstrate that hyperglycaemia, but not hyperlipidaemia, induces the redox-regulator p66^Shc^ and reactive oxygen species (ROS) in macrophages. p66^Shc^ expression, ROS generation, and a pro-atherogenic phenotype are sustained despite restoring normoglycemic conditions. Inhibition of p66^Shc^ abolishes this sustained pro-atherogenic phenotype, identifying p66^Shc^-dependent ROS in macrophages as a key mechanism conveying the hyperglycaemic memory. The p66^Shc^-associated hyperglycaemic memory can be reversed by aPC via protease-activated receptor-1 signalling. aPC reverses glucose-induced CpG hypomethylation within the p66^Shc^ promoter by induction of the DNA methyltransferase-1 (DNMT1). Thus, epigenetically sustained p66^Shc^ expression in plaque macrophages drives the hyperglycaemic memory, which—however—can be reversed by aPC. This establishes that reversal of the hyperglycaemic memory in diabetic atherosclerosis is feasible.

## Abstract (web summary)

Diabetic atherosclerosis fails to improve even when the blood glucose level returns to normal. Shahzad et al. show that this hyperglycaemic memory is linked with epigenetically sustained expression of the redox-regulator p66Shc in plaque-macrophages, which can be corrected by activated protein C.

## Introduction

Loss of thrombomodulin expression and impaired activated protein C (aPC) generation have been associated with atherosclerosis and diabetes mellitus^1–6^. Plaque morphology differs among diabetic and non-diabetic atherosclerotic disease, indicating at least partially disjunct pathophysiology^7,8^. Indeed, despite intensive lipid lowering the probability of atherosclerosis and myocardial infarction remains increased in diabetic patients, suggesting that diabetes-specific mechanisms contribute to atherosclerosis independent of elevated blood lipids in hyperglycaemic patients^9,10^. In striking contrast to the improvement of atherosclerotic disease following lipid lowering in humans and mice^11,12^, diabetes-associated atherosclerosis is perpetuated despite marked improvement of blood glucose control^13^. The sustained disease process despite improved blood glucose is referred to as the hyperglycaemic memory^14,15^. The hyperglycaemic memory is linked with glucose-induced post-translational modifications and epigenetically sustained gene expression^16,17^. Approaches to therapeutically reverse the hyperglycaemic memory are lacking.

The protein p66^Shc^, which can translocate to the outer mitochondrial space and interact with the electron-transport chain, resulting in increased mitochondrial ROS generation^18^, has been linked with glucose-induced excess ROS generation and diabetes-associated vascular complications^16,19^. Glucose-induced p66^Shc^ expression has been observed in podocytes (renal epithelial cells), endothelial cells, and smooth muscle cells^16,19,20^. Expression of p66^Shc^ is epigenetically controlled, suggesting that sustained p66^Shc^ expression may provide a mechanistic link between the hyperglycaemic memory, sustained vascular ROS generation and inflammation, and progressive atherosclerotic disease despite improved blood glucose control^16,19^. A recent study demonstrated sustained p66^Shc^ expression in peripheral blood monocytes of diabetic patients despite improved blood glucose control^21^. However, whether sustained p66^Shc^ expression likewise occurs in atherosclerotic plaque-associated macrophages remains unknown. Likewise, it remains unknown whether p66^Shc^ expression in macrophages is causatively linked with diabetes-associated atherosclerosis and whether targeting p66^Shc^ expression may provide a therapeutic benefit. We previously reported that aPC normalizes glucose-induced p66^Shc^ expression in podocytes^16^, suggesting that impaired aPC generation in the context of diabetes mellitus may contribute to sustained p66^Shc^ expression and thus to the hyperglycaemic memory in diabetic patients. Here we demonstrate that aPC reverses the glucose-induced, epigenetically sustained p66^Shc^ expression in atherosclerotic plaque-associated macrophages, thus promoting the reversal of hyperglycaemia-induced atherosclerotic plaques.

## Results

### Hyperglycaemia promotes plaque instability in ApoE^−/−^ mice

To gain insights into specific pathomechanisms of hyperglycaemia versus hyperlipidaemia-induced atherosclerotic plaque development we directly compared hyperglycaemic (induced by low-dose streptozotocin, STZ injection for 5 days, a model of type 1 DM) and hyperlipidaemic (induced by a high-fat diet, HFD) ApoE^−/−^ mice with control ApoE^−/−^ mice. Mice were followed up for 22 weeks. As expected, body weight, blood lipids, or blood glucose levels differed among treatment groups (Supplementary Fig. 1).

**Fig. 1:**
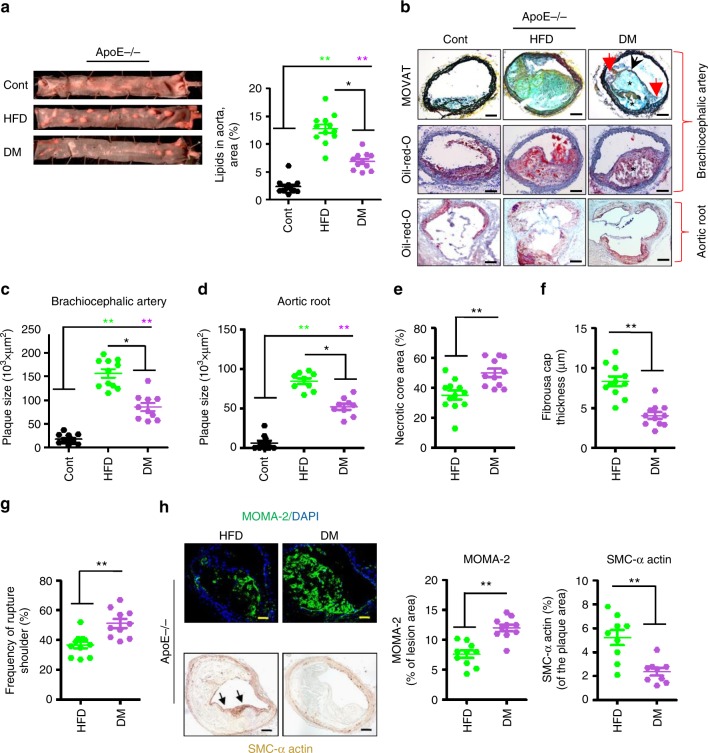
Plaques are less stable in hyperglycaemic versus hyperlipidaemic ApoE-deficient mice. **a** Representative images of thoracic aortae showing en face plaques as detected by Oil Red O staining (left panel) and dot plot summarizing data (total area stained with Oil Red O, right panel) in control (normal chow diet, Cont), hyperlipidaemic (high-fat diet, HFD), and diabetic (STZ-induced hyperglycaemia, DM) mice. **b**–**g** Representative images showing MOVATs staining (**b**, upper panel) and Oil Red O staining of brachiocephalic arteries (**b**, middle panel) and aortic roots (**b**, lower panel). Morphometric analyses of MOVATs and Oil Red O-stained images reveal smaller plaques (**c**, **d**), but increased necrotic core area (**e**), thinner fibrous caps (**f**), and more ruptured shoulders (**g**) in ApoE^−/−^ DM mice compared to ApoE^−/−^ HFD. Dot plots in **e**–**g** summarize results obtained from MOVAT-stained brachiocephalic arteries (**b**, top). Necrotic core area within plaque is indicated by **b** *, fibrous caps thickness by a black arrow, and ruptured shoulders by a red arrow (**b**). **h** Representative images showing immunofluorescence staining of macrophages (upper panel, MOMA-2, green; DAPI nuclear counterstain, blue) and smooth muscle cells (lower panel, α-actin, positive cells detected by HRP-DAB reaction, brown) within lesions and dot plot summarizing data. Cont: non-diabetic ApoE^−/−^ mice receiving normal chow diet; HFD: ApoE^−/−^ mice receiving high-fat diet (HFD); DM: hyperglycaemic ApoE^−/−^ mice. Data shown as dot plots represent mean ± SEM of 10–12 mice per group (**a**, **c**–**h**); size bars: **b**, **h**: 20 µm; *P < 0.05, **P < 0.01; **a**, **c**, **d**: one-way ANOVA with Bonferroni-adjusted post hoc comparison of HFD and DM versus Cont and HFD versus DM; **e**–**h** *t*-test

Analyses of Oil Red O-stained aortae en face revealed an increase of lipid deposits in ApoE^−/−^ HFD mice compared to ApoE^−/−^ DM mice (Fig. 1a). Likewise, plaques within the brachiocephalic artery (Fig. 1b, c) and the aortic roots (Fig. 1b, d) were larger in ApoE^−/−^ HFD than in ApoE^−/−^ DM mice. To evaluate whether the reduced plaque size in hyperglycaemic mice simply reflects the lower total plasma cholesterol levels, we compared a subgroup of ApoE^−/−^ DM and ApoE^−/−^ HFD mice matched for total plasma cholesterol levels (922 ± 52 mg dl^−1^ versus 912 ± 46 mg dl^−1^ for HFD and DM mice, respectively). Despite matching a subgroup of ApoE^−/−^ HFD and ApoE^−/−^ DM mice for total plasma cholesterol levels lesion size was still smaller in hyperglycaemic ApoE^−/−^ DM mice compared to ApoE^−/−^ HFD mice (Supplementary Fig. 2). The smaller plaque size in ApoE^−/−^ DM mice suggests that the aggravated disease course of hyperglycaemia-related atherosclerosis is not primarily related to an increased plaque size. Hence, we next evaluated cellular composition and other parameters reflecting plaque stability.

Indeed, signs of plaque instability were more frequent in ApoE^−/−^ DM mice than in ApoE^−/−^ HFD mice. Thus, plaques in ApoE^−/−^ DM mice had an increased necrotic core area (Fig. 1e), thinner fibrous caps (Fig. 1f), and an increased frequency of ruptured plaque shoulders (Fig. 1g). Plaque morphology and stability depend in part on cellular composition. In plaques of ApoE^−/−^ DM mice macrophages area (immunohistochemically positive for MOMA-2) were increased, while smooth muscle cells (SMC α-actin-positive area) were reduced in comparison to plaques of ApoE^−/−^ HFD mice (Fig. 1h). The observed shift to more macrophages and less SMC corroborates reduced plaque stability in ApoE^−/−^ DM mice.

### Hyperglycaemia induces p66^Shc^ and CD36 in macrophages

**Fig. 2:**
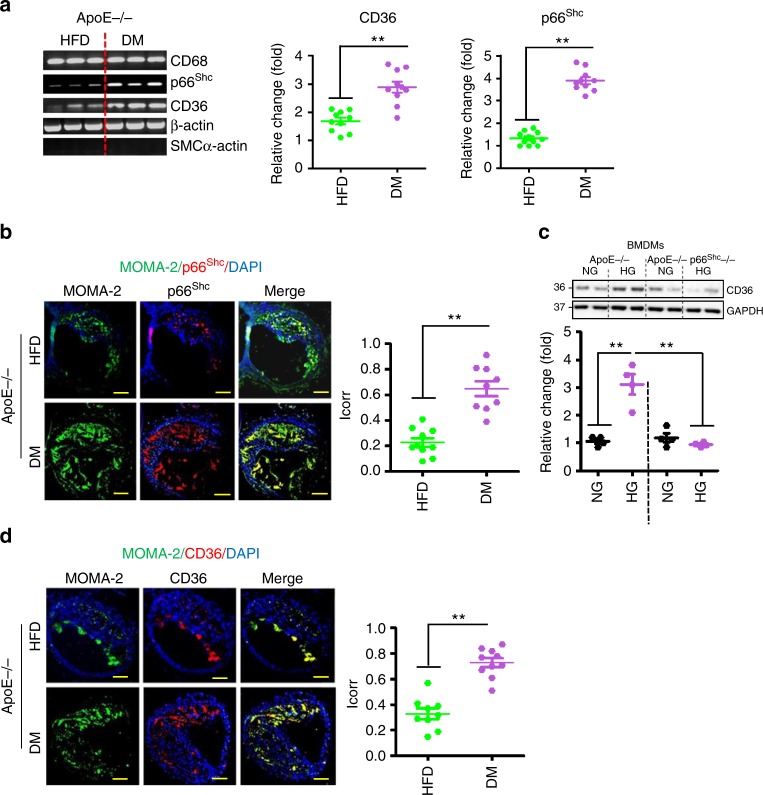
p66^Shc^ and CD36 expressions are increased in plaque-associated macrophages in diabetic mice. **a**, **b** Representative reverse-transcriptase-PCR gel images showing expression of CD68, p66^Shc^, CD36, β-actin (loading control), and SMCα-actin (reflecting purity of laser-dissected macrophages, **a**, left panel) and dot plot summarizing data for CD36 (**a**, middle panel) and p66^Shc^ (**a**, right panel). Representative images showing co-immunofluorescence staining for p66^Shc^ (red), MOMA-2 (green), and DAPI nuclear counterstain (blue) within lesions of the brachiocephalic artery (**b**, left panel) and dot plot summarizing data using automated digital co-localization analyses, yielding a correlation index (*I*_corr_) (**b**, right panel). **c** Representative immunoblot images of CD36 (out of 4 independent repeat experiments with two technical replicates each, top) and dot plot summarizing data (bottom) in ApoE^−/−^ and ApoE^−/−^ p66^Shc−/−^ bone marrow-derived macrophages (BMDMs); NG normal glucose (5 mM), HG high glucose (25 mM), GAPDH: loading control. **d** Representative co-immunofluorescence images for CD36 (red), MOMA-2 (green), DAPI nuclear counterstain (blue) within lesions of the brachiocephalic artery in ApoE^−/−^ HFD and ApoE^−/−^ DM mice (left panel) and dot plot summarising data (*I*_corr_, correlation index, right panel). DM: hyperglycemic ApoE^−/−^ mice; HFD: ApoE^−/−^ mice with high-fat diet (HFD). Data shown as dot plots represent mean ± SEM of 10 mice per group (**a**, **b**, **d**) or 4 biological distinct replicates (**c**); size bars: **b**, **d**: 20 µm; **P < 0.01; **a**, **b**, **d**: *t*-test; **c**: two-way ANOVA with Bonferroni-adjusted post hoc comparison of ApoE^−/−^ (NG, HG) versus p66^Shc−/−^ ApoE^−/−^ (NG, HG, respectively) BMDMs. Uncropped reverse-transcriptase PCR gel images for Fig. 2a and immunoblots for Fig. 2c are provided in Supplementary Figure 19

Plaque-associated macrophages impair plaque stability in part by generating reactive oxygen species (ROS). We therefore analysed expression of the redox-regulator p66^Shc^ in plaque-associated macrophages of ApoE^−/−^ DM and ApoE^−/−^ HFD mice. Expression of p66^Shc^ was markedly enhanced in CD68-positive laser-dissected plaque macrophages of ApoE^−/−^ DM mice (Fig. 2a and Supplementary Fig. 3). Immunohistochemical analyses confirmed increased p66^Shc^ expression and co-localization of p66^Shc^ with MOMA-2, another macrophage marker, in plaques of ApoE^−/−^ DM mice (Fig. 2b).

As ROS induces expression of the scavenger receptor CD36, we speculated that enhanced p66^Shc^ expression increases CD36 expression in hyperglycaemic mice. First, we ascertained whether p66^Shc^ conveys glucose-induced CD36 expression. ApoE^−/−^ or ApoE^−/−^ p66^Shc^-deficient (ApoE^−/−^ p66^Shc−/−^) mouse bone marrow-derived macrophages (BMDMs) were cultured under normoglycaemic (5 mM glucose plus 20 mM mannitol, NG) or hyperglycaemic (25 mM glucose, HG) conditions for 48 h. HG conditions-induced CD36 expression in ApoE^−/−^, but not in ApoE^−/−^ p66^Shc−/−^ BMDMs (Fig. 2c), demonstrating that p66^Shc^ mediates the glucose-dependent induction of CD36 in macrophages. Consistently, CD36 expression was markedly increased in laser-dissected macrophages obtained from plaques of ApoE^−/−^ DM mice (Fig. 2a). Immunohistochemical analyses verified an increased expression and co-localization of CD36 in plaque-associated macrophages of ApoE^−/−^ DM versus ApoE^−/−^ HFD mice (Fig. 2d).

**Fig. 3:**
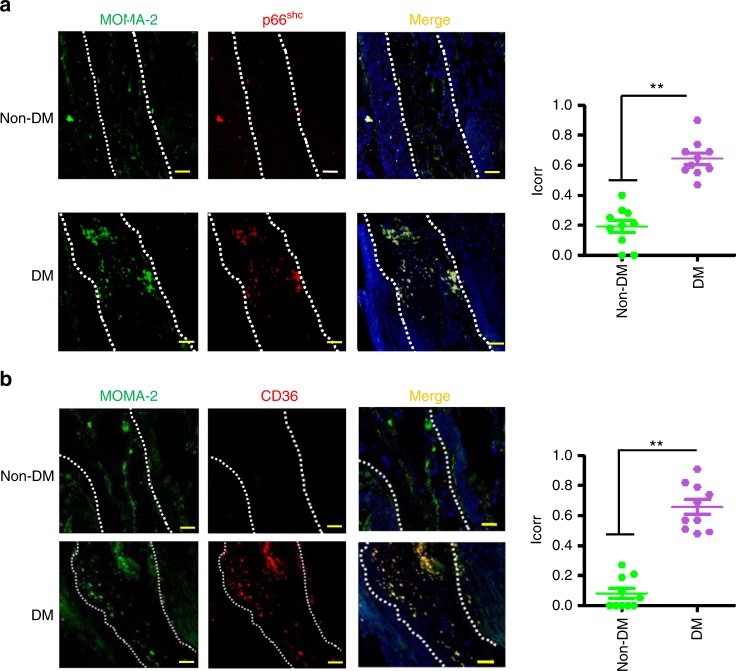
p66^Shc^ and CD36 expressions are increased in plaque-associated macrophages in diabetic patients. Representative co-immunofluorescence images for p66^Shc^ (red, **a**, left panel), CD36 (red, **b**, left panel), MOMA-2 (green, **a**, **b**, left panels), and DAPI nuclear counterstain (blue) and dot plot summarising data (*I*_corr_; correlation index, right panels). Co-localization of p66^Shc^ and CD36 with the macrophage marker MOMA-2 is increased in diabetic patient’s samples (*N* = 10) as compared to non-diabetic patient’s samples (*N* = 10). DM: diabetic patient’s samples; non-DM: non-diabetic patient’s samples. Data shown as dot plots represent mean ± SEM of 10 patients per group, size bars: 20 µm; **P < 0.01; **a** Mann–Whitney *U* test, **b** *t*-test

To investigate the potential translational relevance, we analysed p66^Shc^ and CD36 expression in atherosclerotic plaques of non-diabetic and diabetic patients. As in mice, both p66^Shc^ and CD36 expressions were more abundant in atherosclerotic plaques of diabetic patients compared to those of non-diabetic patients (Fig. 3). Again, p66^Shc^ and CD36 expression was predominately observed in macrophages (co-localization of p66^Shc^ and MOMA-2, Fig. 3). Thus, in diabetic mice and humans, macrophages within atherosclerotic plaques express more p66^Shc^ and CD36 compared to non-diabetic mice or humans.

### p66^Shc^ is crucial for hyperglycaemia-induced atherosclerosis

**Fig. 4:**
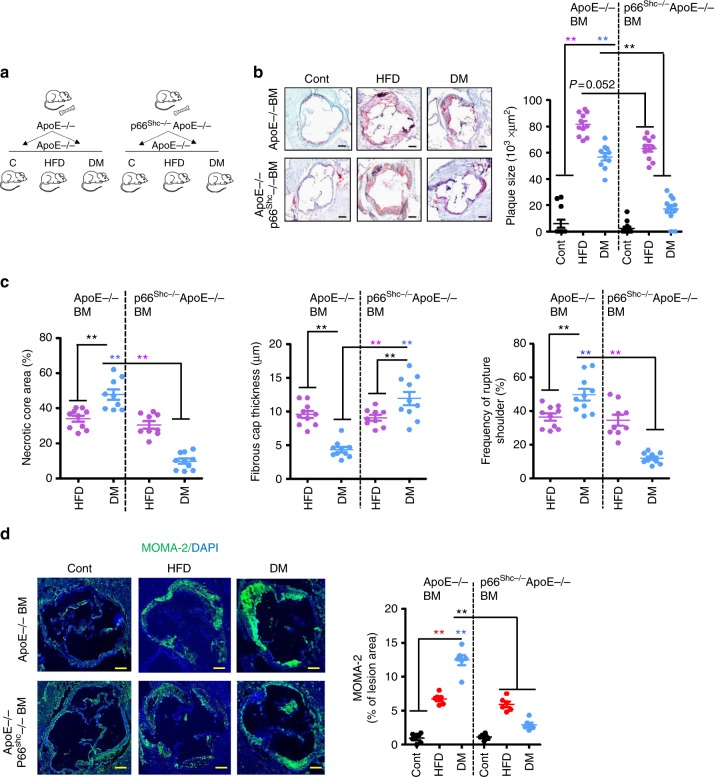
p66^Shc^ has a pivotal function in hyperglycaemia-, but not hyperlipidaemia-induced atherosclerosis. **a**. Experimental design of bone marrow transplantation experiments. **b**. Transplantation of p66^Shc−/−^ deficient bone marrow markedly reduces hyperglycaemia-, but only slightly hyperlipidaemia-induced atherosclerosis. Representative images showing Oil Red O staining of aortic roots (left panel) and dot plot summarizing plaques size (right panel). **c**. Dot plots summarizing results of necrotic core area (left panel), fibrous cap thickness (middle panel), and frequency of ruptured shoulders (right panel). **d**. Representative images showing immunofluorescence staining of macrophages (MOMA-2, green; DAPI nuclear counterstain, blue) within lesions (left panel) and dot plot summarizing data (right panel). Cont: normoglycaemic ApoE^−/−^ mice with normal chow diet; DM: hyperglycaemic ApoE^−/−^ mice; HFD: ApoE^−/−^ mice with high-fat diet (HFD). Data shown as dot plots represent mean ± SEM of 6–10 mice per group (**b**–**d**); size bars: **b**, **d**: 20 µm; **P < 0.01; **b**–**d**: two-way ANOVA with Bonferroni-adjusted post hoc comparison of ApoE^−/−^ (Cont, HFD, DM) versus p66^Shc−/−^ ApoE^−/−^ (Cont, HFD, DM respectively) recipient mice

To evaluate the pathogenic relevance of p66^Shc^ in myeloid-derived cells for hyperglycaemia-associated atherosclerosis we conducted bone marrow transplantation experiments. Bone marrow isolated from ApoE^−/−^ or p66^Shc−/−^ApoE^−/−^ mice was transplanted into lethally irradiated ApoE^−/−^ mice (age 8 weeks, Fig. 4a). Control mice were left untreated (control), while in experimental mice hyperglycaemia or hyperlipidaemia were induced at age 10 weeks. As expected, body weight, total plasma cholesterol, or blood glucose levels differed among groups, but transplantation of ApoE^−/−^ or p66^Shc−/−^ApoE^−/−^ derived bone marrow had no impact on these parameters (Supplementary Fig. 4).

Following transplantation of p66^Shc−/−^ApoE^−/−^ derived bone marrow plaque size was reduced, (Fig. 4b). However, the reduction in plaque size was less prominent and only of borderline significance (*P* = 0.052) in the HFD group, while a pronounced reduction of plaque size was apparent in the DM group (Fig. 4b). Indeed, plaque size in p66^Shc−/−^ApoE^−/−^ transplanted ApoE^−/−^ DM mice was comparable to that in the controls (Fig. 4b). The markedly reduced plaque size in p66^Shc−/−^ApoE^−/−^ transplanted ApoE^−/−^ DM mice was associated with a decreased necrotic core area, increased fibrous cap thickness, decreased frequency of rupture shoulder, and less plaque-associated macrophages. (Fig. 4c, d), reflecting increased plaque stability. Thus, hyperglycaemia-associated impaired plaque stability primarily depends on p66^Shc^ expression in myeloid-derived cells.

**Fig. 5:**
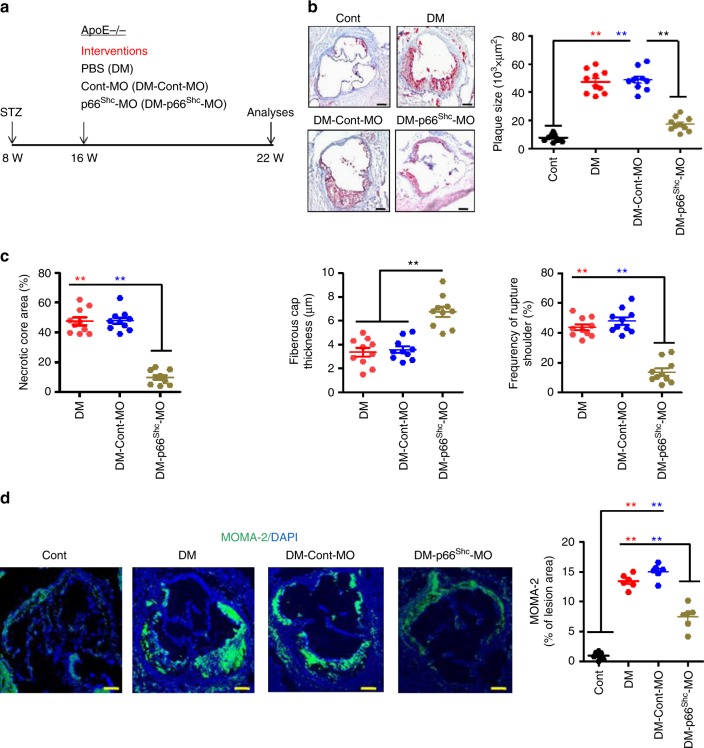
In vivo silencing of p66^Shc^ by vivo morpholino reduces hyperglycaemia-induced atherosclerosis. **a** Experimental design of vivo morpholino p66^Shc^ silencing experiments. **b** Representative images of Oil Red-O staining of the aortic root lesions (left panel) and dot plot summarizing plaque size (right panel). **c** Dot plots summarizing result of necrotic core area (left panel), fibrous cap thickness (middle panel), and frequency of ruptured shoulders (right panel). **d** Representative images showing immunofluorescence staining of macrophages (MOMA-2, green; DAPI nuclear counterstain, blue) within lesions (left panel) and dot plot summarizing data (right panel). Cont: normoglycaemic control mice; DM: hyperglycaemic ApoE^−/−^ mice treated with PBS; Cont-MO: hyperglycaemic ApoE^−/−^ mice treated with control vivo morpholino; p66^Shc^-MO: hyperglycaemic ApoE^−/−^ mice treated with p66^Shc^-specific vivo morpholino. Data shown as dot plots represent mean ± SEM of 6–10 mice per group (**b**–**d**); size bars: **b**, **d** 20 µm; **P < 0.01; one-way ANOVA with Bonferroni-adjusted post hoc comparison of DM and DM-Cont-MO versus Cont and DM-p66^Shc^-MO versus DM-Cont-MO (**b**, **d**) or DM-p66^Shc^-MO versus DM-Cont-MO and DM (**c**)

To determine whether p66^Shc^ may be a therapeutic target in diabetes-associated atherosclerosis we treated mice with vivo morpholinos (VM) to repress p66^Shc^ expression (p66^Shc^-MO). After 16 weeks of persistent hyperglycaemia ApoE^−/−^ DM mice were injected with p66^Shc^-MO (DM-p66^Shc^-MO) or control-MO (DM-Cont-MO; Fig. 5a). Efficient suppression of p66^Shc^ in the aorta was confirmed by semi-quantitative reverse-transcriptase PCR (Supplementary Fig. 5a) and immunofluorescence staining (Supplementary Fig. 5b). Treatment with VM had no impact on body weight, blood glucose, or blood lipid levels (Supplementary Fig. 6). Following suppression of p66^Shc^ plaques within aortic roots (Fig. 5b) and brachiocephalic arteries (Supplementary Fig. 7) were smaller in p66^Shc^-MO as compared to Cont-MO-treated ApoE^−/−^ DM mice. This was associated with increased signs of plaque stability as reflected by decreased necrotic core area, increased fibrous cap thickness, decreased frequency of rupture shoulder and less plaque-associated macrophages in p66^Shc^-MO as compared to Cont-MO-treated ApoE^−/−^ DM mice (Fig. 5c, d). These results demonstrate that p66^Shc^ expression in macrophages promotes diabetes-accelerated atherosclerosis and that targeting p66^Shc^ may be a feasible therapeutic approach in diabetes-associated atherosclerosis.

### Sustained p66^Shc^ and CD36 expression in macrophages

**Fig. 6:**
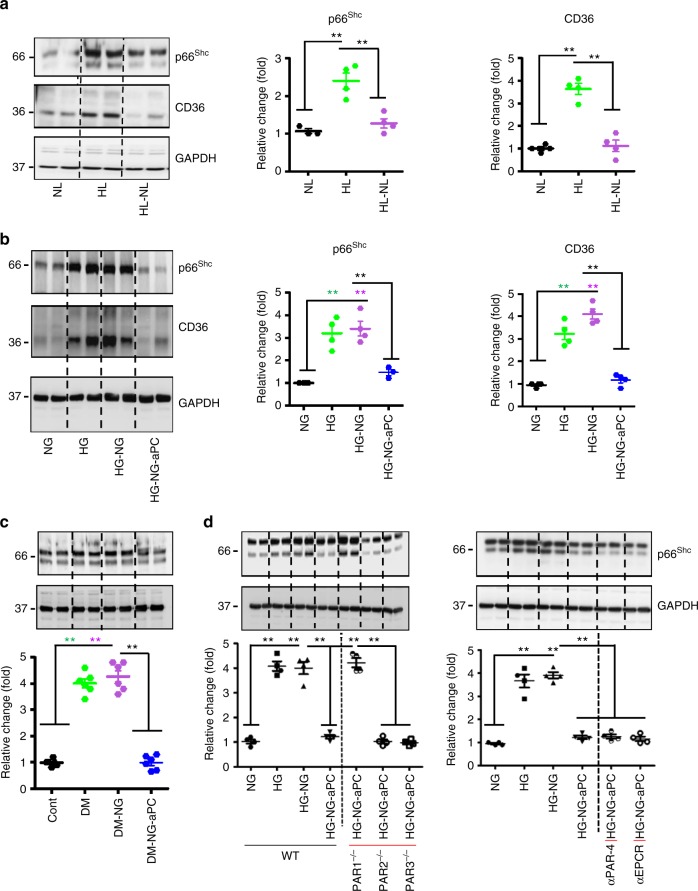
aPC reverses glucose-induced sustained p66^Shc^ expression and the pro-atherogenic phenotype of macrophages. **a**, **b** Representative immunoblot images (out of four independent repeat experiments with two technical replicates each, left) and dot plot summarizing relative expression levels (right panels) of p66^Shc^, CD36, and GAPDH (loading control) in mouse bone marrow-derived macrophages (BMDMs) exposed to different lipid (**a**) or glucose (**b**) concentrations without or with additional aPC treatment. **c** Representative immunoblot images (out of 6 independent repeat experiments with two technical replicates each, top) and dot plot summarizing data (bottom) of p66^Shc^ and GAPDH (loading control) in BMDMs isolated from non-hyperglycaemic (control, Cont), hyperglycaemic (DM, 22 weeks persistent hyperglycaemia), transiently hyperglycaemic (DM-NG, 16 weeks of sustained hyperglycaemia followed by 6 weeks of SGLT2-inhbitor treatment), or DM-NG-aPC (DM-NG mice with aPC treatment parallel to SGLT2-inhibitors treatment). **d** Representative immunoblot images of p66^Shc^ (out of four independent repeat experiments with two technical replicates each) and GAPDH (loading control) in wild-type (WT), PAR1^−/−^, PAR2^−/−^, or PAR3^−/−^ BMDMs (**d**, left panel) and in WT-BMDMs exposed to inhibitory antibodies of PAR4 or EPCR (**d**, right panels). NL: normal LDL; HL: high LDL (50 µg/ml, 48 h); HL-NL: 50 µg/ml LDL, 48 h, followed by NL, 24 h); NG: normal glucose (5 mM glucose plus 20 mM mannitol, NG, 48 h); HG: high glucose (25 mM, 48 h); HG-NG: high glucose followed by normal glucose (25 mM glucose, 48 h, followed by 5 mM glucose, 24 h), HG-NG-aPC: HG-NG treated in addition with aPC (20 nM for the last 24 h); Data shown as dot plots represent mean ± SEM of at least 3 independent repeat experiments with at least two technical replicates each; **P < 0.01; one-way ANOVA with Bonferroni-adjusted post hoc comparison of HL versus NL and HL-NL versus HL (**a**), HG and HG-NG versus NG and HG-NG-aPC versus HG-NG (**b**, **d**), DM and DM-NG versus Cont and DM-NG-aPC versus DM-NG (**c**), HG-NG-aPC (αPAR4 and αEPCR-4 BMDMs) versus HG-NG-aPC (**d**, right panel) BMDMs. One-way ANOVA with Bonferroni-adjusted post hoc comparison of WT (HG and HG-NG versus NG and HG-NG-aPC versus HG-NG) and HG-NG-aPC (PAR3^−/−^ and PAR2^−/−^ BMDMs) versus HG-NG-aPC (PAR1^−/−^ BMDMs) (**d**, left panel). Uncropped immunoblots for Fig. 6a–d are provided in Supplementary Figure 20 and Supplementary Figure 21, respectively

To evaluate whether glucose induces persistent p66^Shc^ expression in macrophages we exposed mouse BMDMs to high LDL (50 µg/ml, HL) or high glucose (25 mM, HG) for 48 h in vitro. High LDL (but not low LDL, Fig. 6a) induced p66^Shc^ and CD36 expression. Similarly, high glucose-induced (but not mannitol, Fig. 6b) p66^Shc^ and CD36 expression. We then ascertained whether normalization of glucose or lipid concentration reverses elevated p66^Shc^ and CD36 expression in BMDMs. Following normalization of LDL for additional 24 h (HL-NL) p66^Shc^ and CD36 expression returned to baseline (Fig. 6a). In contrast, despite normalization of glucose concentration for the last 24 h (HG-NG) p66^Shc^ and CD36 expression remained elevated in BMDMs (Fig. 6b). Thus, high glucose causes a sustained induction of p66^Shc^ and CD36 expression in macrophages in vitro.

### aPC reverses sustained p66^Shc^ expression via PAR1

We next determined whether aPC reverses p66^Shc^ expression in glucose-stressed macrophages in vitro. BMDMs were left untreated or exposed for 48 h to high glucose concentrations, followed by normal glucose concentrations for 24 h (HG-NG). Concomitant aPC treatment (20 nM, HG-NG-aPC) during the 24 h period of normalized glucose concentrations markedly reduced p66^Shc^ expression as compared to PBS-treated controls (HG-NG, Fig. 6b). Parallel changes were observed for CD36 expression, indicating that glucose-induced persistent CD36 expression depends on p66^Shc^, but can be normalized by aPC (Fig. 6b).

To evaluate the in vivo relevance of these findings we analysed freshly isolated BMDMs, which were in vivo conditioned using different treatment schemes: (A) control mice (Cont., no hyperglycaemia), (B) diabetic mice (DM, 22 weeks of persistent hyperglycaemia), and (C) diabetic mice, in which blood glucose levels were normalized for 6 weeks after 16 weeks of persistent hyperglycaemia using the SGLT2-inhbitor dapagliflozin (DM-NG). The DM-NG group was randomly assigned to receive either PBS (DM-NG-PBS) or aPC (DM-NG-aPC) parallel to the treatment with the SGLT2-inhbitor (Supplementary Fig. 8a). aPC treatment had no impact on blood glucose levels (Supplementary Fig. 8b). Expression of p66^Shc^ was high in BMDMs derived from DM and DM-NG-PBS mice, but markedly reduced in DM-NG-aPC mice (Fig. 6c), demonstrating that aPC normalizes glucose-induced sustained p66^Shc^ expression not only in vitro, but also in vivo.

To identify the receptors through which aPC reduces p66^Shc^ expression in glucose stressed BMDMs we first determined the expression of protease-activated receptors (PARs) and endothelial protein C receptor (EPCR) in BMDMs. Expression of PAR1, PAR2, PAR3, PAR4, and EPCR was readily detectable in BMDMs (Supplementary Fig. 9). To determine the functional relevance of these receptors we isolated BMDMs from PAR1^−/−^, PAR2^−/−^, or PAR3^−/−^ mice, while PAR4 or EPCR function was blocked in wild-type BMDMs using inhibitory antibodies. PAR1 deficiency efficiently abolished p66^Shc^ inhibition by aPC, whereas PAR2, PAR3 deficiency or PAR4 and EPCR inhibition had no effect (Fig. 6d). Hence, PAR1 is required for the aPC-dependent inhibition of glucose-induced sustained p66^Shc^ expression in BMDMs.

### aPC reverses the sustained atherogenic macrophage phenotype

**Fig. 7:**
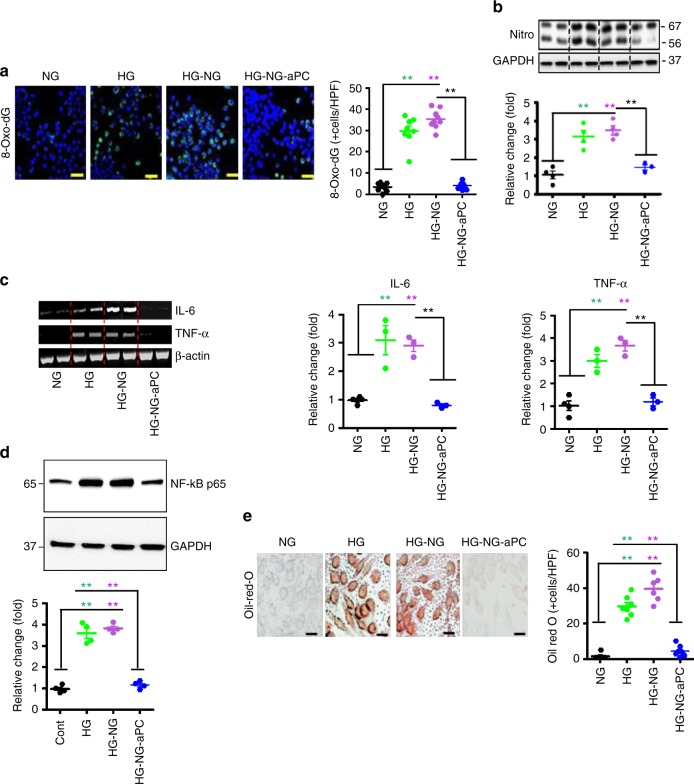
aPC reverses the glucose-induced and sustained pro-atherogenic phenotype of macrophages. **a**, **b** Representative co-immunofluorescence images (**a**, left panel) for 8-Oxo-dG (green; DAPI nuclear counterstain, blue) and immunoblots images (out of three independent repeat experiments with two technical replicates each) for nitrotyrosine (Nitro, **b**, top; GAPDH: loading control) of BMDMs with treatment as indicated and dot plot summarizing data (**a**, right panel, **b**, bottom). **c** Representative reverse-transcriptase-PCR gel images (out of three independent repeat experiments with two technical replicates each) for IL-6 and TNF-α (right panel; β-actin: loading control) and dot plot summarizing data (middle and right panels, respectively). **d** Representative immunoblot images (out of four independent repeat experiments with two technical replicates each) for NF-κB p65 (top panel; GAPDH: loading control) and dot plot summarizing data (bottom). **e** Representative Oil Red O images reflecting lipid uptake into BMDMs (left panel) and dot plot summarizing data (right panel). NG: normal glucose (5 mM glucose plus 20 mM mannitol, 48 h), HG: high glucose (25 mM, 48 h), HG-NG: HG (48 h) followed by NG (24 h) condition; HG-NG-aPC: HG-NG conditions with additional exposure to aPC (20 nM) during the last 24 h). Data shown as dot plots represent mean ± SEM of at least three independent repeat experiments with at least two technical replicates each; **P < 0.01 (one-way ANOVA with Bonferroni-adjusted post hoc comparison of HG and HG-NG versus NG and HG-NG-aPC versus HG-NG). Uncropped immunoblots for Fig. 7b, d and reverse-transcriptase-PCR gel images for Fig. 7c are provided in Supplementary Figure 22

To determine whether aPC modulates p66^Shc^ dependent functional consequences in macrophages we analysed ROS and inflammatory markers. In HG exposed BMDMs 8-Oxo-dG (8-Oxo-2’-deoxyguanosine) and nitrotyrosine, both reflecting ROS generation, were induced and remained high despite normalization of glucose levels (HG-NG, Fig. 7a, b). The sustained expression of these ROS-markers was reversed by concomitant aPC treatment (HG-NG-aPC, Fig. 7a, b). In parallel, IL-6, TNF-α, and NF-κB p65 expression were induced in BMDMs and remained high despite normalization of glucose levels, but were reversed by concomitant aPC treatment (HG-NG-aPC, Fig. 7c, d and Supplementary Fig. 10). In agreement with the observed CD36 expression pattern lipid uptake by BMDMs was increased by HG and remained high despite normalization of glucose levels (HG-NG, Fig. 7e), but was normalized by aPC (HG-NG-aPC, Fig. 7e). Thus, glucose induces sustained pro-inflammatory and pro-atherogenic effects in macrophages in vitro, which can be efficiently reversed by aPC.

### aPC epigenetically inhibits sustained p66^Shc^ expression

**Fig. 8:**
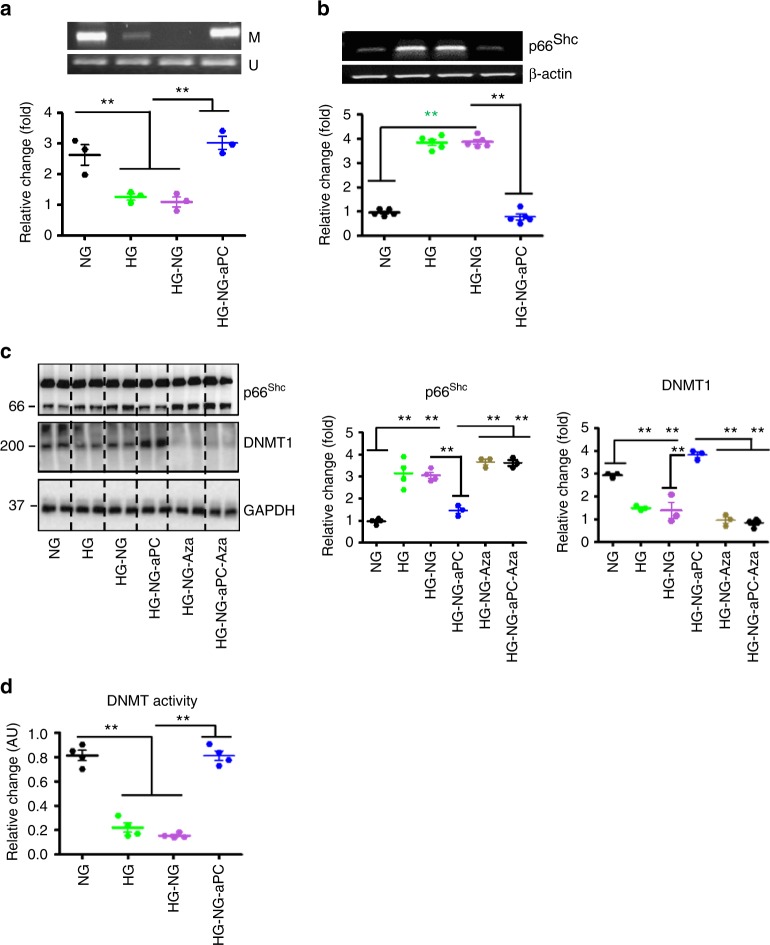
aPC epigenetically inhibits glucose-induced sustained p66^Shc^ expression. **a** Representative reverse-transcriptase-PCR gel images (out of three independent repeat experiments, top) showing methylated (M) and unmethylated (U) p66^Shc^ promoter DNA in BMDMs with treatment as indicated and dot plot summarizing the ratio of methylated to unmethylated p66^Shc^ promoter DNA (bottom). **b** Glucose-induced sustained p66^Shc^ mRNA expression is prevented by aPC (20 nM) in BMDMs cells. Representative RT-PCR gel image (out of five independent repeat experiments, top; β-actin: loading control) and dot plot summarizing data (bottom). **c** Representative immunoblot images (out of 3 independent repeat experiments with two technical replicates each) for p66^Shc^ and DNMT1 expression in BMDMs with treatment as indicated (left panel; GAPDH: loading control) and dot plot summarizing data (middle and right panels). **d** dot plot summarizing DNMT activity in BMDMs with treatment as indicated. NG: normal glucose (5 mM glucose plus 20 mM mannitol, 48 h), HG: high glucose (25 mM, 48 h). HG-NG: HG (24 h) followed by NG (24 h) condition; HG-NG-aPC: HG-NG conditions with additional exposure to aPC (20 nM) during the last 24 h); HG-NG-Aza: HG-NG conditions with additional exposure to the DNMT-inhibitor 5-azacytidine (Aza, 5 µM) during the last 24 h; HG-NG-aPC-Aza: HG-NG conditions with additional and concomitant exposure to aPC (20 nM) and 5-azacytidine (5 µM) during the last 24 h. Data shown represent mean ± SEM of at least three independent experiments each with at least two technical replicates (**a**–**d**); **P < 0.01; one-way ANOVA with Bonferroni-adjusted post hoc comparison of HG and HG-NG versus NG, HG-NG-aPC versus HG-NG (**a**, **b**, **d**), and HG-NG, HG-NG-Aza, HG-NG-aPC-Aza versus DM-NG-aPC). Uncropped reverse-transcriptase-PCR gel images for Fig. 8a, b and immunoblots for Fig. 8c are provided in Supplementary Figure 23

We next analysed p66^Shc^ promoter methylation in macrophages. Compared to control BMDMs (normal glucose, NG) CpG dinucleotides within the p66^Shc^ promoter were hypomethylated in BMDMs maintained under hyperglycaemic conditions (HG, Fig. 8a). The p66^Shc^ promoter remained hypomethylated despite normalization of glucose levels for 24 h (HG-NG, Fig. 8a). However, concomitant aPC treatment restored p66^Shc^ promoter methylation (Fig. 5a). Corresponding changes were observed for p66^Shc^ mRNA levels (Fig. 8b).

To gain further mechanistic insights we determined DNMT expression parallel to p66^Shc^ expression in BMDMs. The expression pattern of DNMT1 was opposite to that of p66^Shc^ (Fig. 8c), while DNMT3a and DNMT3b expression remained unchanged (Supplementary Fig. 11). We observed concomitant changes in DNMT activity (Fig. 8d). Of note, the DNMTs-inhibitor 5-azacytidine (Aza) abolished the inhibitory effect of aPC on glucose sustained p66^Shc^ expression (HG-NG-aPC-Aza, Fig. 8c). This suggests that the induction of DNMT1 by aPC is required for aPC-mediated repression of sustained p66^Shc^ expression.

### Sustained p66^Shc^ impairs atherosclerosis regression

We next evaluated whether glucose-induced epigenetic p66^Shc^ expression contributes to the hyperglycaemic memory in macrovascular disease and whether this can be reversed by aPC. First, we established a “memory effect” in glucose, but not in lipid exposed ApoE^−/−^ mice (see Experimental scheme, Supplementary Fig. 12a). Aortic root plaque size was prominently reduced following lowering of blood lipid levels in HFD-adeno hApoE versus HFD-adeno LacZ mice (Supplementary Fig. 12b-e). Conversely, the size of atherosclerotic plaques remained unchanged following restoration of normoglycaemia (Supplementary Fig. 12b–e), reflecting sustained plaques size secondary to the hyperglycaemic memory.

**Fig. 9:**
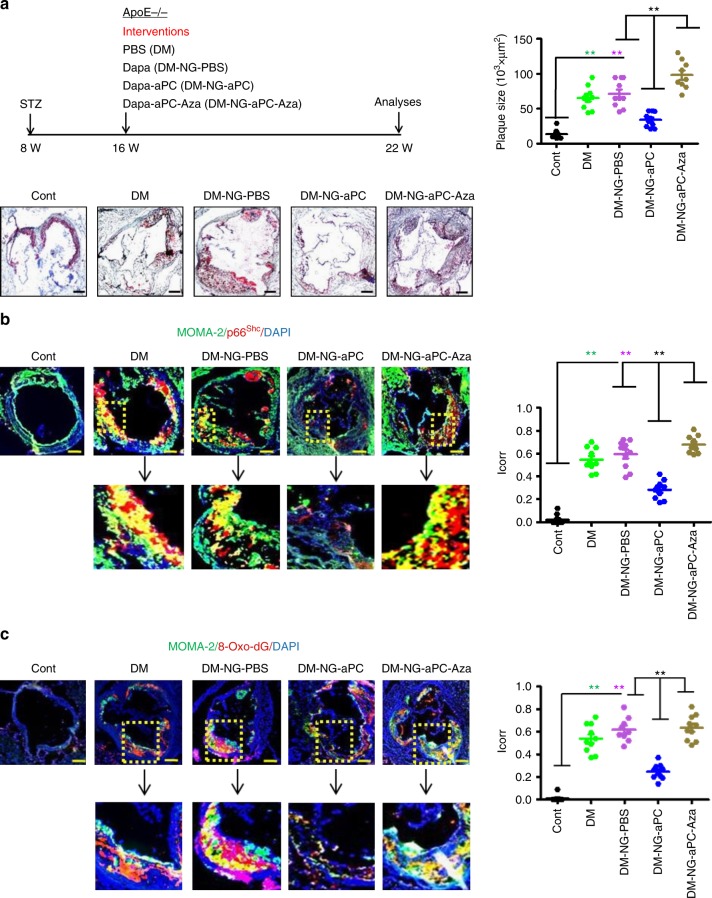
aPC reverses hyperglycaemic induced and epigenetically sustained p66^Shc^ expression, promoting atherosclerotic plaque regression in mice. **a** Experimental design (left top) and representative Oil Red O-stained images of aortic roots (left bottom) and dot plot summarizing data (right). **b**, **c** Representative co-immunofluorescence images for p66^Shc^ (**b**, red), 8-Oxo-dG (**c**, red), macrophages (MOMA-2, **b**, **c**, green), and DAPI nuclear counterstain (**b**, **c**, blue; boxed areas shown at higher magnification in the lower panel). Dot plot summarize data for p66^Shc^-MOMA-2 (**b**) and 8-Oxo-dG-MOMA-2 co-localization (**c**, both *I*_corr_, correlation index). Cont: normoglycaemic ApoE^−/−^ mice with normal chow diet; DM: hyperglycaemic ApoE^−/−^ mice; DM-NG-PBS: ApoE^−/−^ DM mice receiving the SGLT2 inhibitor and PBS from week 16 to week 22; DM-NG-aPC: ApoE^−/−^ DM mice with concomitant SGLT2 inhibitor and aPC treatment from week 16 to week 22; DM-NG-aPC-Aza: DM-NG mice with concomitant SGLT2 inhibitor, aPC, and 5-azacytidine. Data shown as dot plots represent mean ± SEM of 8–10 mice per group (**a**, **c**); size bars: **a**–**c**: 20 µm; **P < 0.01 (one-way ANOVA with Bonferroni-adjusted post hoc comparison of DM and DM-NG-aPC versus control and DM-NG-PBS and DM-NG-aPC-Aza versus DM-NG-aPC)

Using this model, we were able to determine whether aPC reverses the hyperglycaemic memory effect in the context of atherosclerosis in vivo. Indeed, concomitant aPC treatment in addition to blood glucose reduction markedly reduced the size of aortic root plaques when compared to mice in which only blood glucose levels were normalized (DM-NG-aPC versus DM-NG-PBS, Fig. 9a). Furthermore, concomitant aPC treatment (DM-NG-aPC) decreased necrotic core area, the frequency of rupture shoulders, plaque-associated macrophages, while increasing fibrous cap thickness (Supplementary Fig. 13a,b). Importantly, concomitant aPC treatment reduced p66^Shc^ expression, ROS generation (8-Oxo-dG), and IL-6 expression, while normalization of blood glucose levels alone had no impact on these parameters (Fig. 9b, c and Supplementary Fig. 13c,d). Treatment of mice with 5-azacytidine in addition to aPC treatment and blood glucose normalization (DM-NG-aPC-Aza) abolished aPC’s effects (Fig. 9 and Supplementary Fig. 13). Treatment with aPC or 5-azacytidine had no impact on body weight, blood glucose levels, or total plasma cholesterol levels (Supplementary Fig. 14).

Taken together, these data demonstrate that aPC in addition to normalization of blood glucose levels reverses the high glucose-induced sustained p66^Shc^ expression and atherosclerotic plaque phenotype in ApoE^−/−^ mice.

### aPC reverses diabetes-associated atherosclerosis via DNMT1

**Fig. 10:**
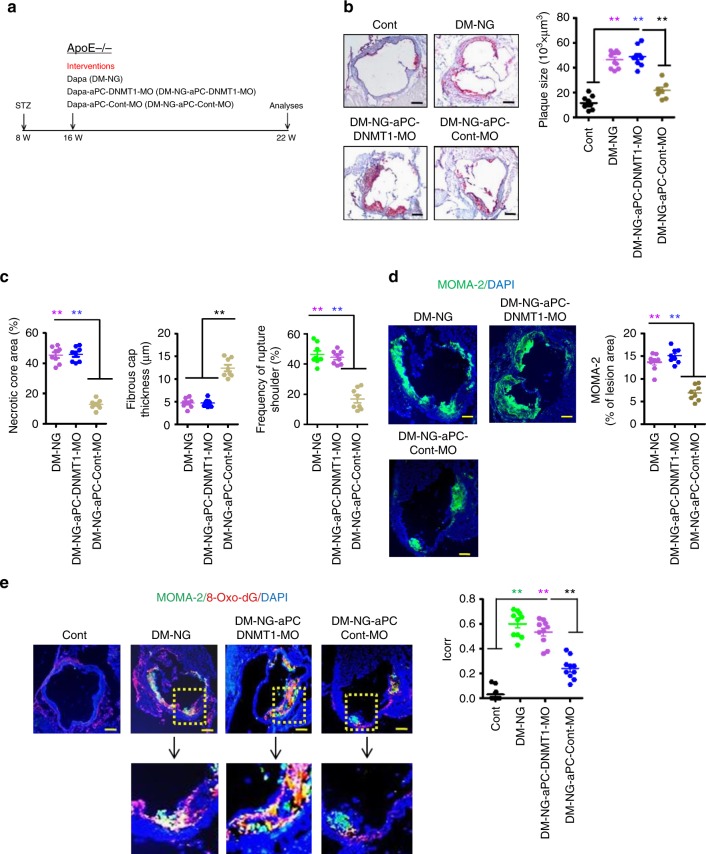
aPC-mediated reversal of hyperglycaemia-induced persistent p66^Shc^ expression and plaque instability depends on DNMT1. **a** Experimental design. **b** Representative images of Oil Red O-stained aortic root lesions (left panel) and dot plot summarizing data (right panel). **c** Dot plots summarizing morphometric analyses of necrotic core area (left panel), fibrous cap thickness (middle panel), and frequency of ruptured shoulders (right panel). **d** Representative images showing immunofluorescence staining of macrophages within lesions (MOMA-2, green; DAPI nuclear counterstain, blue; left panel) and dot plot summarizing data (right panel). **e** Representative co-immunofluorescence images for 8-Oxo-dG (red), macrophages (MOMA-2, green), and DAPI nuclear counterstain (blue) within aortic root lesions (left, ×10 magnified images shown in lower panel) and dot plot summarizing data (*I*_corr_, correlation index, right). Cont: normoglycaemic ApoE^−/−^ mice with normal chow diet; DM-NG: ApoE^−/−^ DM mice receiving the SGLT2 inhibitor and PBS from week 16 to week 22; DM-NG-aPC-DNMT1-MO: DM-NG mice with concomitant SGLT2 inhibitor, aPC, and DNMT1-MO treatment; DM-NG-aPC-Cont-MO: DM-NG mice with concomitant SGLT2 inhibitor, aPC, and control morpholino treatment. Data shown as dot plots represent mean ± SEM of 8–10 mice per group; size bars: **b**, **d**, **e** 20 µm; **P < 0.01; **b**–**e** one-way ANOVA with Bonferroni-adjusted post hoc comparison of DM-NG and DM-NG-aPC-DNMT1-MO versus DM-NG-aPC-Cont-MO)

We next determined the in vivo relevance of DNMT1 for the aPC-mediated reversion of sustained p66^Shc^ expression in macrophages. Expression of DNMT1 was reduced in the aorta of diabetic (DM) animals and remained low despite normalization of blood glucose levels for 6 weeks after 16 weeks of persistent hyperglycaemia (DM-NG, Supplementary Fig. 15). Concomitant treatment with aPC restored DNMT-1 expression (DM-NG-aPC, Supplementary Fig. 15). Since constitutive genetic DNTM1 deficiency is lethal in mice, we inhibited DNMT1 expression in diabetic ApoE^−/−^ mice using VM (DNMT1-MO, Fig. 10a). ApoE^−/−^ mice with hyperglycaemia (DM) for 16 weeks followed by 6 weeks of normoglycaemia (NG, achieved by SGLT2 inhibitor treatment) and concomitant aPC treatment were injected with a DNMT1 specific morpholinos (DM-NG-aPC-DNMT1-MO) or a non-specific control morpholino (DM-NG-aPC-Cont-MO, Fig. 10a). The DNMT1 specific morpholino sufficiently reduced DNMT1 expression in the aorta (DM-NG-aPC-DNMT1-MO), while a control morpholino (DM-NG-aPC-Cont-MO) had no impact (Supplementary Fig. 16a). Suppression of DNMT1 expression abolished the effect of aPC, as reflected by a failure of aPC to reduce plaque size (Fig. 10b) or to improve plaque stability, as reflected by an unchanged necrotic core area, fibrous cap thickness, frequency of rupture shoulder (Fig. 10c), and frequency of plaque-associated macrophages (Fig. 10d) in DM-NG-aPC-DNMT1-MO mice as compared to DM-NG mice. (Fig. 10c, d). Likewise, aPC failed to reduce 8-Oxo-dG levels, p66^Shc^ expression, and IL-6 expression in the aorta (Fig. 10e and Supplementary Fig. 16b, c). Treatment with VM had no impact on body weight, blood glucose, or total plasma cholesterol levels (Supplementary Fig. 17). Thus, the aPC-mediated reversal of the hyperglycaemic memory phenotype in hyperglycaemic ApoE^−/−^ mice depends on DNMT1.

## Discussion

Reduced thrombomodulin expression and aPC plasma levels have been clinically associated with diabetes and its vascular complications^2–4,6^. While impaired thrombomodulin-dependent protein C activation has been mechanistically linked with diabetic microangiopathy^1,16,22,23^, the role of aPC in diabetic macroangiopathy remained unknown. Here we demonstrate that aPC reverses the hyperglycaemic memory by correcting glucose-induced p66^Shc^ promoter hypomethylation in macrophages via a DNMT1-dependent mechanism. Thus, aPC in addition to blood glucose normalization reduces the atherosclerotic plaque burden, signs of plaque instability, and associated inflammation. Importantly, the current data do not only identify a mechanism contributing to the hyperglycaemic memory in atherosclerosis (glucose-induced and DNMT1-dependent sustained p66^Shc^ expression in macrophages), but also identifies a potential therapeutic approach to the atherosclerosis-associated hyperglycaemic memory. The current data suggest that targeting p66^Shc^ expression in macrophages either directly (as demonstrated by the VM approach) or indirectly by employing aPC-based therapies is a feasible approach to combat the hyperglycaemic memory in atherosclerosis^24,25^.

By directly comparing hyperglycaemic ApoE^−/−^ (ApoE^−/−^ DM) mice to non-diabetic ApoE^−/−^ mice on a high-fat diet (ApoE^−/−^ HFD) we first show that the expression of the redox-regulator p66^Shc^ in bone marrow-derived cells is a major determinant of plaque development and instability in hyperglycaemic conditions. While normalization of blood glucose levels alone fails to reduce glucose-induced and epigenetically sustained p66^Shc^ expression in macrophages, aPC in addition to blood glucose normalization reverses glucose-induced p66^Shc^ promoter hypomethylation and p66^Shc^ expression in macrophages and associated ROS generation. These aPC-mediated consequences are congruent with aPC’s anti-oxidant effects and its effect on p66^Shc^ expression in glucose stressed podocytes (renal epithelial cells)^16,26^.

We specifically addressed the consequences of persistently elevated glucose level and associated changes for the hyperglycaemic memory in the current manuscript. The results show that hyperglycaemia per se drives plaque instability in murine models of hyperglycaemia-associated atherosclerosis. These findings add to previous important work, demonstrating that insulin resistance promotes unstable plaques through chronic inflammation^27–29^. Hence, we propose that insulin resistance and hyperglycaemia both promote plaque instability, and that the concurrence of insulin resistance and hyperglycaemia in insulin resistance type 2 diabetic patients may synergistically promote plaque instability. Accordingly, we cannot exclude that other metabolic changes, such as obesity, increased insulin levels, or impaired insulin signaling, contribute to a “memory” effect in type 2 diabetic patients. Indeed, epigenetic control of p66^Shc^ in association with obesity has been recently reported^30^. Here the methyltransferase SUV39H1 was identified as a key regulator modulating p66^Shc^ expression by orchestrating recruitment of JMJD2C (a demethylase) and SRC-1 (an acetyltransferase) to the p66^Shc^ promoter^30^. Intriguingly, SUV39H1 forms a complex with DNMT1^31^, suggesting that SUV39H1 and DNMT1 co-ordinately modulate the epigenetic landscape controlling p66^Shc^ expression. Further characterization of the complex modulating the p66^Shc^-associated epigenetic landscape and thus p66^Shc^ expression may identify additional therapeutic targets. This may lay ground for individualized treatment strategies in the context of diabetes-associated atherosclerosis, as recently proposed^32^.

The translational relevance of the current finding is supported by a recent clinical study which demonstrated that p66^Shc^ expression is epigenetically increased in peripheral blood monocytes of type 2 diabetic patients^21^. In agreement with these clinical results we observed increased p66^Shc^ expression in plaque-associated macrophages of diabetic, but not of non-diabetic patients and mice. The concordant observation in human peripheral blood monocytes^21^, human plaque-associated macrophages (Fig. 3), and the murine data presented within this manuscript argue against sustained p66^Shc^ expression in ApoE^−/−^DM mice as a consequence of toxic streptozotocin effects^33^. Congruently, we were not able to detect increased cell death, markers of the DNA damage response, or liver toxicity in STZ-injected ApoE^−/−^ mice. Taken together, these studies identify p66^Shc^ as an epigenetically controlled gene modulating the phenotype of monocytes and macrophages in diabetes. We speculate that the p66^Shc^ dependent modulation of the macrophage phenotype constitutes a diabetes-specific facet of trained innate immunity^34,35^. aPC-based approaches may allow modulation of trained innate immunity in the context of diabetes.

The pivotal role of p66^Shc^ in bone marrow-derived cells, the co-localization of p66^Shc^ with macrophages, and the expression of pro-atherogenic (CD36) and pro-inflammatory (NF-κB p65, IL-6, and TNFα) genes in hyperglycaemia-associated macrophages are entirely congruent with the previously proposed pathogenetic function of macrophages in diabetes-associated atherosclerosis in animal^36–38^ and clinical studies^39^. In extension of previous work we thus identify p66^Shc^ as a specific regulator of diabetes-associated atherosclerosis^40^. Different pathways through which increased and sustained p66^Shc^ expression may impair plaque stability, such as increased macrophages cell death, enhanced ROS-induced inflammasome activation, or impaired efferocytosis may contribute to impaired plaque stability in hyperglycaemic mice. Similar to p66^Shc^, ACSL-1 (long-chain acyl-CoA synthetase 1, an enzyme that catalyzes the thioesterification of fatty acids) promotes atherosclerotic plaque development specifically in a diabetic milieu^41^. Collectively, these studies support the concept of a specific pathophysiology underlying diabetes-associated atherosclerosis, thus providing a rationale for “individualized” therapeutic strategies in patients with diabetes-associate atherosclerosis.

Hyperglycaemia is thought to cause mitochondrial dysfunction, thus promoting diabetes-associated vascular complications^42^. The role of p66^Shc^ for diabetic vascular complications, as shown here and previously^16,19,43^, supports a pivotal role of mitochondrial ROS generation. Excess ROS generation activates the redox-sensitive transcription factor NF-κB, promoting a pro-inflammatory micromilieu characterized by increased IL-6, TNFα, CCL2, or VCAM-1 expression^44^. Intriguingly, inhibition of pro-inflammatory NF-κB signalling by aPC has been demonstrated before^45,46^, but the underlying mechanism remained unknown. Given the current insights we propose that aPC restricts p66^Shc^-mediated ROS generation and thus NF-κB signalling. Additionally, increased ROS generation promotes NO-uncoupling and inflammasome activation, which are closely linked with diabetic vascular complications^47^. The observed induction of IL-6 and TNFα in glucose stressed macrophages is entirely congruent with these observations and with the perception of diabetic vascular complications as inflammation driven diseases^48,49^. The current data indicate that endothelial dysfunction with impaired thrombomodulin protein C activation accelerates and perpetuates the hyperglycaemia-induced vascular inflammation by unrestrained p66^Shc^-mediated ROS generation in macrophages.

Other mechanisms of increased ROS generation have been linked with diabetes-associated accelerated atherosclerosis^50^. Intriguingly, enhanced p66^Shc^ expression induces NADPH oxidase activity, while repressing manganese superoxide dismutase (MnSOD) expression, resulting in increased ROS generation in peripheral blood monocytes^51^. Accordingly, enhanced p66^Shc^ expression may be a common pathomechanism inducing mitochondrial and other mechanisms of increased ROS generation, thus generating a pro-inflammatory and pro-atherogenic micromilieu.

Mitochondrial dysfunction in bone marrow-derived cells promotes unstable plaques^42^. Here we show that p66^Shc^ (a mitochondrial redox regulator) promotes CD36 expression and lipid uptake in glucose-stimulated macrophages. As excess lipid uptake promotes cell death and thus the evolution of an unstable acellular—or necrotic—core, increased and perpetuated p66^Shc^ expression may contribute to the impaired plaque stability observed in diabetic patients^10^.

While providing insights, the current study has potential limitations. We aimed to directly compare mice with hyperlipidaemia and hyperglycaemic associated atherosclerosis. However, due to the study design total plasma cholesterol levels and plasma HDL levels were not comparable between groups. While a subgroup analyses of ApoE^−/−^ DM and ApoE^−/−^ HFD mice gave similar results (Supplementary Fig. 2), we cannot exclude an impact of different plasma levels of lipid species such as HDL cholesterol. Yet, we are confident that the observed changes largely reflect hyperglycaemia-dependent effects as the endpoints analysed (e.g., p66^Shc^ expression and its sequelae: ROS generation, CD36, IL-6, TNFα-expression) were increased in the hyperglycaemic milieu compared to the hyperlipidaemic milieu despite lower total cholesterol levels in the hyperglycaemic milieu.

Of note, the mixed phenotype in diabetic ApoE^−/−^ mice, characterized by hyperglycaemia and hyperlipidaemia, reflects the situation in diabetic patients, in which likewise both risk factors are increased. Accordingly, some consider the ApoE^−/−^ streptozotocin model to be the most appropriate mouse model to study accelerated diabetes-associated atherosclerosis^52^. The recent clinical observation showing epigenetically sustained p66^Shc^ expression in diabetic patients^21^ and the increased p66^Shc^ expression in atherosclerotic lesions from diabetic as compared to non-diabetic patients in the current study supports the translational relevance of the model used.

Finally, the question arises how the current finding may be taken further. Approaches to directly target p66^Shc^ may be appropriate in addition to exploiting aPC-based signalling. In the current study morpholinos targeting p66^Shc^ efficiently restricted hyperglycaemia-induced lesion development. Targeting morpholinos to relevant cell types, e.g., macrophages, through a receptor-dependent mechanism, e.g., via CD36, may constitute an approach to enable a targeted therapy, thus avoiding unwanted site effects. Alternatively, biased PAR1 agonists may be suitable to restrict p66^Shc^ expression in plaque macrophages. These questions need to be addressed in future studies.

## Methods

### Reagents

The following antibodies were used in the current study: rabbit anti-p66^Shc^ (Merck, Millipore, United States); rabbit anti-CD36, mouse anti-nitrotyrosine, rabbit anti-PAR4 (Santa Cruz, Germany); rabbit anti-DNMT1, rabbit anti-NF-κB p65, rabbit anti-DNMT3a rabbit anti-DNMT3b, rabbit anti-β-actin, goat anti-rabbit IgG HRP (Cell Signaling Technology, Germany); mouse anti-8-hydroxy-2’-deoxyguanosine (Trevigen, United States); rat anti-MOMA-2, rabbit anti-mouse IgG HRP (abcam, Germany); rat anti-endothelial cell protein C receptor (EPCR), rabbit anti-GAPDH (Sigma-Aldrich, Germany). The following secondary antibodies for immunofluorescence were used: FITC goat anti-rabbit IgG, FITC rabbit anti-goat IgG, Texas red rabbit anti-mouse IgG, and FITC donkey anti-goat IgG (Vector Laboratories, United States).

Other reagents were as follows: DMEM, trypsin-EDTA, penicillin, streptomycin, FCS, FBS, and HEPES (PAA Laboratories, Austria); BCA reagent (Perbio Science, Germany); vectashield mounting medium with DAPI (Vector Laboratories, United States); PVDF membrane and immobilon^TM^ western chemiluminescent HRP substrate (Merck, Millipore, United States); streptozotocin (Enzo Life Sciences, Germany); saffron, Oil Red-O, L929 cell line (Sigma-Aldrich, Germany); Accu-Chek test strips, Accu-Check glucometer, protease inhibitor cocktail (Roche Diagnostics, Germany); albumin fraction V, hematoxylin Gill II, acrylamide, agarose (Carl ROTH, Germany); aqueous-mounting medium (ZYTOMED, Germany); human LDL (Alfa Aesar, Germany); “high-fat diet” (HFD) experimental food (Western Type Diet, 43% carbohydrates, 15% proteins and 42%, Ssniff, Germany); Trizol Reagent and PBS (Life Technologies, Germany); RevertAid™ H Minus First Strand cDNA Synthesis kit (Fermentas, Germany); rompun 2% (Bayer, Germany); ketamine 10% (beta-pharm, Germany).

### Mice

ApoE^−/−^ (002052), PAR2^−/−^ (024161), PAR3^−/−^ (015230) mice were obtained from the Jackson Laboratory (Bar Harbor, ME, USA). PAR1^−/−^ mice were provided by Eric Camerer (Paris, France) and p66^Shc−/−^ mice have been described previously^16,18,47^. C57BL/6J were obtained from Janvier (S.A.S., St. Berthevin Cedex, France). In the current study we used littermates backcrossed for at least ten generations on a C57BL/6J background. Only age-matched mice were used throughout the study. All animal experiments were conducted following standards and procedures approved by the local Animal Care and Use Committee (Landesverwaltungsamt Halle, Germany).

### Atherogenic mouse models

ApoE^−/−^mice (age 6 to 8 weeks) were either fed high-fat diet (HFD) or normal chow diet or were made diabetic (DM) by injecting streptozotocin a model of type 1 DM (STZ, 60 mg kg^−1^, intraperitoneally, once daily for 5 consecutive days, freshly dissolved in 0.05 M sterile sodium citrate, pH 4.5)^16,53^. Control mice were injected with equal volume of 0.05 M sodium citrate, pH 4.5 for 5 days and maintained on a normal chow diet^47^. Blood glucose and body weight were measured once weekly^16,53^. On average 85–90% of mice became diabetic (blood glucose > 300 mg dl^−1^) within the first 4 weeks and these were included as diabetic mice in the experiments. Mice not developing persistently elevated blood glucose levels and maintaining blood glucose levels < 200 mg dl^−1^ despite STZ injections were included in the control group^1^. The endpoints analysed did not differ between STZ-injected, but normoglycaemic mice and sodium-citrated injected mice (controls). HFD or hyperglycaemia (minimum 300 mg/dl) was maintained for up to 22 weeks. In some experiments diabetic mice were treated with the SGLT2 inhibitor dapagliflozin (25 mg kg^−1^ in the drinking water)^53^ 16 weeks after the last STZ injection to reduce blood glucose levels without (DM-NG) or with concomitant aPC (1 mg kg^−1^ intraperitoneally once daily, DM-NG-aPC)^16^ or concomitant aPC plus 5-azacytidine (additionally 0.25 mg kg^−1^ intraperitoneally on alternative days, DM-NG-aPC-Aza) treatment for further 6 weeks. Mice were killed and plaque morphology was analysed as previously described^5^. A group of HFD mice was randomly assigned to (a) persistent hyperlipidaemia (HFD-adeno LacZ) or (b) normalization of blood lipids for 6 weeks after 16 weeks of hyperlipidaemia by re-expressing human ApoE in the liver (HFD-adeno hApoE)^54^. Mice were randomly allocated to different groups. All animal experiments were performed using 6–12 mice per group and data from all mice was included in the study to ensure biological reproducibility of in vivo data.

Atherosclerotic plaque morphology was analysed using the Image Pro Plus software from Media Cybernetics as previously described^5^. Briefly, plaque characteristics were determined as described in the following section (please see also Supplementary Fig. 18): Total plaque size (in μm^2^) is defined as the size of the plaque comprising all parts of the atheroscleroma (fibrous cap, necrotic tissue, fibrous tissue etc.) within the vessel lumen. Necrotic core (in percent) is the area staining blue upon MOVATs stain and is reported as the percentage of the total plaque size. Fibrous cap thickness (in μm) is the minimal thickness of the fibrous tissue overlaying a necrotic core. If multiple necrotic cores are present within one plaque, the thickness of all fibrous caps is determined and the average used for further analyses. Frequency of rupture shoulder (in percent) is defined by the detection of a broken fibrous cap upon MOVATs staining and/or intraplaque haemorrhage, as indicated by intraplaque erythrocytes, and then calculating the number of ruptured shoulders compared to the total shoulder number.

### Vivo Morpholinos oligomer treatment

Morpholinos (MO) were obtained from Gene Tools, LLC, United States^55^. The following oligonucleotides were used: 5’-ATCCCCAGGCCCTTAGCCTAGTCT G-3’ against p66^Shc^ (Mus musculus src homology 2 domain-containing transforming protein C1, Shc1, transcript variant 1), blocking the translation of Shc1 transcript variant 1; 5’- ATCGCCAGGCGCTTAGCGTAGTGTG-3’ as the p66^Shc^ mismatch control; 5’-CAGGTTGCAGACGACAGAACAGCTC-3’ against DNA methyltransferase, DNMT1, transcript variant 1, blocking the translation of DNMT1 transcript variant 1; 5’-CAGCTTCCAGACCACACAACACCTC-3’ as the DNMT1 mismatch control. MO’s were dissolved in PBS (100 μl; 6 mg MO per kg body weight; intravenously) and were injected every other day for 6 weeks into a subset of diabetic ApoE^−/−^ mice.

### Analysis of mice

After 22 weeks of age the mice’s body weight was measured and mice were killed^5,56^. Blood samples were obtained from the inferior vena cava of anticoagulated mice (500 U unfractionated heparin, intraperitoneally). Blood was centrifuged at 2.000 × *g* for 20 min at 4 °C and plasma was snap frozen in liquid nitrogen. Mice were perfused with ice-cold PBS for 10 min and the heart, aortic arches, including brachiocephalic arteries, were embedded in O.C.T. compound and snap frozen. Brachiocephalic arteries were sectioned from distal to proximal at 6 μm thickness. The thoracic aorta was fixed in 4% buffered formalin for 20 min, washed twice in PBS for 10 min and stored for not more than 1 day at 4 °C before analysis.

### Analysis of blood lipids

Blood lipids were measured with the Cobas 6000 Chemistry System from Roche (Basel, Switzerland).

### Laser capture microdissection

Macrophages within atherosclerotic lesions and staining positive for CD68 were laser captured and micro dissected as described elsewhere^57^. RNAse free buffers and solutions were used. Briefly, frozen sections were fixed in cold acetone at 4 °C for 10 min. Then sections were dried at RT for 10 min and washed briefly in 1 × PBS. Sections were then incubated with diluted primary antibody (1:100, rabbit anti-CD68) at RT for 30 min. Afterwards, sections were washed twice with 1 × PBS and incubated with secondary antibody (1:200, Texas red-conjugated anti rabbit) at RT for 30 min. Sections were washed with 1 × PBS and briefly dried at RT in the dark. CD68-positive cells were captured using a laser capture microdissection microscope (PixCell II System, Germany). Cells were captured by placing a cap lined with a thermos labile film onto a thinly cut section of tissue, with “pickup” completed by activating the film with a near-infrared laser diode pulse. The melting of the thermoplastic film caused the cells of interest to adhere to the film, which were then isolated from the surrounding tissue when the cap was lifted away from the slide. RNA was isolated by using the Qiagen RNeasy Micro RNA isolation kit. RNA concentration was measured in a nanometer and a 1.8% agarose gel was run to verify the purity and integrity of RNA. cDNA was synthesized according to manufacturer’s protocol (Sensiscript First-Strand Sythesis System for reverse-transcriptase polymerase chain reaction (RT-PCR) from Qiagen).

### Bone marrow transplantation

ApoE^−/−^ or p66^Shc−/−^ ApoE^−/−^ mice were sacrificed and bones were isolated form the hind limb (tibia, femur) and flushed with medium (RPMI 1640 with 2% FBS, 10 units/ml heparin, penicillin, and streptomycin) using a 25 G needle^47^. To remove osseous particles the solution was passed through a sterile 40 µm nylon Cell Strainer (Falcon) and collected into a 50 ml tube. Cells were centrifuged at 900 × *g* (10 min, 4 °C). Supernatant was discarded and the cell pellet was washed twice with 50 ml of serum-free RPMI (RPMI 1640 with 20 mM Hepes, penicillin, and streptomycin). Following centrifugation at 900 × *g* (5 min, 4 °C) cells were re-suspended in 25 ml of serum-free RPMI. Cell number was determined using a cell counter (TC20^TM^ automated cell counter, Biorad, Germany). Cells were suspended in serum-free RPMI to a final concentration of 1 × 10^7^ cells/ml. Recipient mice (ApoE^−/−^ 8 weeks old) were lethally irradiated (11 Gy, once) and injected with 5 × 10^6^ bone marrow cells (volume 0.2 ml) via the tail vein 4–6 h after irradiation. The mice were kept on antibiotic (sulamethazine; 1 gl^−1^ in the drinking water) for 2 weeks after irradiation and then switched to water without antibiotics. Four weeks after bone marrow transplantation, efficient replacement of bone marrow was ascertained by FACS analysis. Recipient ApoE^−/−^ mice were then divided into three groups: mice received either a high-fat diet (HFD group) or a normal chow diet without (control group) or with persistent hyperglycaemia (DM group). Persistent hyperglycaemia was induced by injecting streptozotocin (60 mg kg^−1^) intraperitoneally once daily for 5 consecutive days, freshly dissolved in 0.05 M sterile sodium citrate, pH 4.5^47,49^. Control mice were injected with equal volume of 0.05 M sodium citrate, pH 4.5 for 5 days and maintained on a normal chow diet^47,49^. After 22 weeks mice were killed and analysed as described above.

### Histology and Immunohistochemistry

Oil Red O staining was conducted on thoracic aortae (opened longitudinally) or frozen sections of the brachiocephalic arteries or aortic roots^5,56^. Tissue were stained with Oil Red O for 10 min, rinsed twice with distilled water for 20 s and once in running tap water for 10 min. Aortae were pinned on a black wax surface using 0.1 mm diameter stainless steel pins as described previously^5,56^. Cryopreserved sections of the brachiocephalic arteries and aortic roots (4 μm) were fixed in ice-cold acetone for 2 min, rinsed twice in ice-cold 1 × PBS, and were stained with Oil Red O using the same protocol as described above. Frozen sections were counterstained with haematoxylin for 40 s, rinsed in tap water, and mounted with aqueous mount. Lipid rich areas of the aortae were analysed by a blinded investigator using the Image Pro Plus software as described previously^5,56^. MOVAT’s stain was performed on frozen sections of brachiocephalic arteries. Frozen sections (4 µm) were fixed in Bouin’s solution at 50 °C for 10 min and stained with 5% sodium thiosulfate for 5 min, 1% alcian blue for 15 min, alkaline alcohol for 10 min, Movat’s Weigert’s solution for 20 min, crocein scarlet acid/fuchsin solution for 1 min, 5 % phosphotungstic acid for 5 min and 1% acetic acid for 5 min. Between every staining step the tissue sections were washed with tap water and distilled water. Afterwards they were dehydrated in 95% and 100% ethanol for 1 min and stained in alcohol saffron for 8 min. Brachiocephalic arteries were washed in 100% ethanol for 1 min, moved to Xylol for 10 min and covered with cytoseal mounting medium. Every 15th section (~90 μm) of the brachiocephalic arteries and aortic roots were analysed to quantify the plaque area. For histological analysis images were captured with an Olympus Bx43-Microscope (Olympus, Hamburg, Gemany). The Image Pro Plus software (version 6.0) software was used for image analysis.

For immunofluorescence frozen sections of brachiocephalic arteries or aortic valves with maximum plaque size were fixed in ice-cold acetone for 8 min, washed twice with ice-cold PBS and incubated in 2% BSA in PBST for 1 h. Sections were then incubated for overnight at 4 °C with one or two primary antibodies against p66^Shc^, DNMT1, CD36, MOMA-2, or 8-Oxo-2’-deoxyguanosine. Sections incubated without primary antibodies were used as negative controls for background correction. After overnight incubation the sections were washed three times with 1 × PBS 5 min each time followed by incubation with fluorescently labelled corresponding secondary antibodies. After washing nuclear counterstaining was conducted using mounting medium with DAPI. Images were visualized, captured, and analysed using a fluorescence microscope. All histological analyses were performed by two independent blinded investigators. Immunohistochemistry and immunofluorescence images were captured with an Olympus Bx43-Microscope (Olympus, Hamburg, Germany). The Image J software was used for image analysis. To determine co-localization of fluorescently detected proteins we determined a correlation index (*I*_corr_). *I*_corr_ reflects the automatically computed fraction (plug in available: https://sites.google.com/site/colocalizationcolormap/home) of positively correlated pixels in an image and is used to quantify colocalized fluorescent signals^58^.

### Isolation and culture of bone marrow-derived macrophages

Bone marrow-derived macrophages (BMDM) were isolated and cultured as described elsewhere^59,60^. Briefly, 10 to 12 weeks old C57BL/6 J mice were sacrificed by cervical dislocation and bones were isolated from hind limbs (tibia, femur). Bones were kept in and flushed with RPMI-1640 complete medium to isolate bone marrow cells. Bone marrow cells were further washed with 1 × PBS and re-suspended in culture medium RPMI supplemented with 30% L929 cell-conditioned medium and 20% FBS. This procedure was repeated twice to remove dead cells. After the final washing step pelleted cells were re-suspended in above culture medium. Cells were cultured for 7 to 10 days until ~80% confluence. The purity of cells was confirmed by CD11b staining and FACS analyses and was consistently found to be higher than 90%. These cells were used as BMDM for experiments.

### Immunoblotting

Proteins were isolated and immunoblotting was performed as described^47,49^. Cell lysates were prepared in RIPA buffer (50 mM Tris at pH 7.4, 1% Nonidet P-40, 0.25% sodium deoxycholate, 150 mM NaCl, 1 mM EDTA, 1 mM Na_3_VO_4_, and 1 mM NaF supplemented with protease inhibitor cocktail). Lysates were centrifuged (10.000 × *g*, 10 min at 4 °C) and insoluble debris was discarded. The protein concentration in supernatants was quantified using BCA reagent. Equal amounts of protein were electrophoretically separated on 7.5%, 10% (vol/vol), or 12.5% (vol/vol) SDS polyacrylamide gels, transferred to PVDF membranes, and probed with the desired primary antibodies overnight at 4 °C. Membranes were then washed with PBS-tween (PBST) and incubated with anti-mouse, anti-rat IgG, or anti-rabbit IgG (each 1:2000) horseradish peroxidase-conjugated antibodies, as indicated. Blots were developed with the enhanced chemiluminescence system. To compare and quantify levels of proteins, the density of each band was measured using ImageJ. Equal loading was confirmed by immuno-blotting with GAPDH antibody.

### Preparation of activated protein C

Activated protein C was generated as previously described^61–63^. Prothrombin complex (Prothromplex NF600), containing all vitamin K-dependent coagulation factors, was reconstituted with sterile water and supplemented with CaCl_2_ at a final concentration of 20 mM. The column for purification of protein C was equilibrated at RT with 1 l of washing buffer (0.1 M NaCl, 20 mM Tris, pH 7.5, 5 mM benzamidine HCl, 2 mM Ca^2+^, 0.02% sodium azide). The reconstituted prothombin complex was gravity eluted on a column filled with Affigel-10 resin covalently linked to a calcium-dependent monoclonal antibody to PC (HPC4). The column was washed first with two column volumes of washing buffer and then two column volumes with a wash buffer rich in salt (0.5 M NaCl, 20 mM Tris, pH 7.5, mM benzamidine HCl, 2 mM Ca^2+^, 0.02% sodium azide). Then the benzamidine was washed off the column with a buffer of 0.1 M NaCl, 20 mM Tris, pH 7.5, 2 mM Ca^2+^, 0.02% sodium azide. To elute PC the column was gravity eluted with elution buffer (0.1 M NaCl, 20 mMTris, pH 7.5, 5 mM EDTA, 0.02% sodium azide, pH 7.5) and 3 ml fractions were collected. The peak fractions were identified by measuring absorbance at 280 nm. The peak fractions were pooled. The recovered PC was activated with human plasma thrombin (5% w/w, 3 h at 37 °C). To isolate activated protein C (aPC) ion exchange chromatography with FPLC (ÄKTAFPLC®, GE Healthcare Life Sciences) was used. First, thrombin was removed with a cation exchange column MonoS (GE Healthcare Life Sciences). Then a MonoQ anion exchange column (GE Healthcare Life Sciences) was equilibrated with 10% of a 20 mM Tris, pH 7.5, 1 M NaCl buffer. After applying the solution that contains aPC a 10–100% gradient of a 20 mM Tris, pH 7.5, 1 M NaCl buffer was run through the column to elute aPC at a flow of 1–2 ml/min under continuous monitoring of OD and conductivity. APC eluted at ~36 mS cm^−1^ by conductivity or at 40% of the buffer. Fractions of 0.5 ml were collected during the peak and pooled. Proteolytic activity of purified aPC was ascertained with the chromogenic substrate SPECTROZYME® PCa.

### Reverse-transcriptase polymerase chain reaction

To isolate RNA from cells 3 ml TRIzol was added to each 10 cm^2^ dish^47,49^. Cells were lysed in TRIzol and incubated for 5 min at RT before adding 0.2 ml of chloroform per 1 ml of TRIzol. Following vigorous shaking the mixture was centrifuged at 12.000 × *g* for 15 min at 4 °C to separate phases. The aqueous phase containing RNA was transferred to a fresh tube and RNA was precipitated by adding 0.5 ml of 2-propanol per 1 ml of TRIzol reagent. Following incubation at RT for 10 min samples were centrifuged at 12.000 × *g* for 10 min at 4 °C. Supernatant was removed and RNA was washed by adding 1 ml 75 % ethanol per 1 ml of TRIzol. Samples were vortexed and centrifuged at 7.500 × *g* for 5 min at 4 °C. The RNA pellet was air-dried for 5 min, redissolved in 20 μl DEPC-water at 55 °C for 10 min. RNA concentration was measured in a nanodrop (2000C, Peq lab, Germany) and a 1.8% agarose gel was run to verify the purity and integrity of RNA. cDNA was synthesized according to manufacturer’s protocol (SuperScript First-Strand Sythesis System for RT-PCR, Fermentas, Germany). Primers were custom synthesized by Thermo Fisher Scientific and PCR was performed using the Taq polymerase. PCR products were separated on a 1.5% (wt/vol) agarose gel and visualized by ethidium bromide staining. Reactions lacking reverse-transcriptase served as negative controls. Primers used in the current study are shown in Supplementary Table 1.

### Methylation-specific PCR

Methylation-specific PCR was performed as previously described^16^. Genomic DNA was isolated using the QIAamp DNA Mini Kit (QIAGEN) and treated with sodium bisulfite using the EZ DNA Methylation Kit (Zymo Research) following the manufacturer’s instructions. PCR amplification was performed with the EpiTect MSP Kit (Qiagen) using primer pairs designed to specifically detect either methylated or unmethylated CpG sites in the mouse p66Shc promoter. For ratio analyses, amplicons against methylated and unmethylated CpGs were visualized on a 1.8% agarose gel. Universal methylated mouse DNA (Zymo research) was used as a positive control. The sequences of the primers used were as follows: methylated forward, 5′-TTT TTT TGG TTT GTT TAC GTC-3′; methylated reverse, 5′-GAC GCG AAA AAA AAA TAA AA-3′; unmethylated forward, 5′-TGT TTT TTT TGG TTT GTT TAT GTT-3′; and unmethylated reverse, 5′-CCA ACA CAA AAA AAA AAT AAA AA-3′.

### DNMT activity assay

To determine DNMTs activity nuclear proteins were extracted from whole cell lysates as described^53^. Culture medium was removed from cells and cells were washed with 1 × PBS and afterwards scrapped in 400 µl of cytoplasmic extraction buffer A containing 10 mM HEPES-KOH (pH 7.9), 10 mM KCl, 1.5 mM MgCl2, 1 mM EDTA, 0.6% NP-40, 0.5 mM DTT, protease inhibitor cocktail. Lysates were kept on ice for 10 min. Lysates were briefly vortexed and then centrifuged at 4 °C. Supernatants containing the cytoplasmic protein fraction were discarded. The pellet was re-suspended in 100 µl nuclear extraction buffer B containing 10 mM HEPES-KOH (pH 7.9), 25% glycerol, 420 mM NaCl, 1.5 mM MgCl2, 0.2 mM EDTA, 0.5 mM DTT and protease inhibitors. Lysates were incubated for 20 min on ice followed by centrifugation at 13.000 × *g* at 4 °C for 5 min. Supernatants containing the nuclear extracts were collected and DNMT activity was measured by using EpiQuik™ DNMT Activity assay ultra kit (EPIGENETEK) following the manufacturer’s instructions.

### Analyses of human samples

Diabetes was diagnosed in patients according to the American Diabetes Association criteria^64,65^. Diabetic (*N* = 10) and non-diabetic (*N* = 10) patients with atherosclerotic disease were recruited from the cardiology clinic at the University Hospital Magdeburg. All 10 diabetic patients were type 2 diabetic patients (T2DM). All patients and controls were Caucasian. All patients were newly admitted to the university hospital at the Otto-von-Guericke University, Magdeburg, for treatment of ACI-stenosis, which was henceforth the primary diagnosis in all patients. Detailed information about the patient’s clinical characteristics is given in Supplementary Table 2. Tissue biopsies of atherosclerotic plaques were obtained from internal carotid artery during carotid disobliteration. Samples were immediately embedded in O.C.T. compound and snap frozen. Tissue biopsies were sectioned at 6-μm thickness and used for immunofluorescence staining as described above. The study complied with the Declaration of Helsinki and all patients entered the study according to the guidelines of the local ethics committees after giving informed consent (Ethic-Committee-No: 92/09).

### Statistical analysis

The data are summarized as mean ± standard error of the mean (SEM). Statistical analyses were performed with Student *t*-test (two-sided), Mann–Whitney *U* test, or analysis of variance (ANOVA), as appropriate. Post hoc comparisons of ANOVA were corrected with the method of Bonferroni, as indicated in figure legends. The Kolmogorov–Smirnov test or D’Agostino-Pearson normality test was used to determine whether the data is consistent with Gaussian distribution. StatistiXL (www.statistixl.com) and Prism 5 (www.graphpad.com) software were used for statistical analyses. Values of *P* < 0.05 were considered statistically significant.

### Data availability

The authors declare that the data supporting the findings of this study are available within the article and its Supplementary Information.

## Electronic supplementary material

Supplementary Information
