# Supplementary material for: Activated protein C reverses epigenetically sustained p66Shc expression in plaque-associated macrophages in diabetes
Source: Commun Biol. 2018 Aug 6;1:104. doi: 10.1038/s42003-018-0108-5 (PMC6123684; doi:10.1038/s42003-018-0108-5)
Supplement: Supplementary file 1 — Supplementary Information [file 42003_2018_108_MOESM1_ESM.pdf]

Supplementary Information

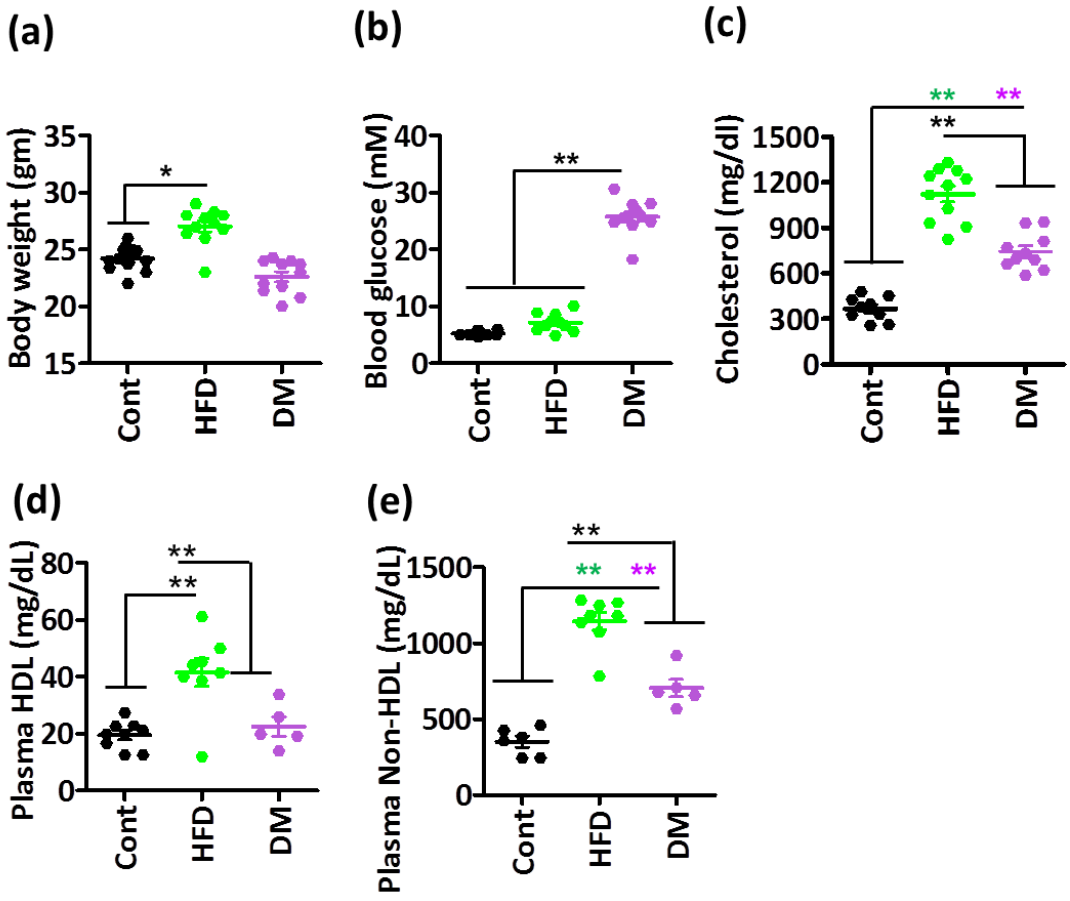

Supplementary Figure 1. Body weight and blood glucose levels in ApoE-/- HFD versus ApoE-/- DM mice (corresponding to Figure 1).

Body weight (**a**), blood glucose levels (**b**), total plasma cholesterol levels (**c**), plasma HDL (**d**) and Non-HDL levels (**e**) in ApoE-/- control mice (Cont, normal chow diet, citrate instead of streptozotocin injections), ApoE-/- HFD mice (high fat diet, no streptozotocin), and ApoE-/- DM mice (normal chow diet, streptozotocin injections). Body weight and total plasma cholesterol levels are increased in ApoE-/- HFD mice as compared to ApoE-/- control mice. In ApoE-/- DM mice body weight is slightly but non-significantly reduced while blood glucose and plasma cholesterol levels are increased.

Data shown as dot-plots represent mean ± SEM of 10-11 mice per group; $^{**}P<0.01$,* $P<0.05$; one-way ANOVA with Bonferroni adjusted post-hoc comparison of HFD and DM versus Cont.

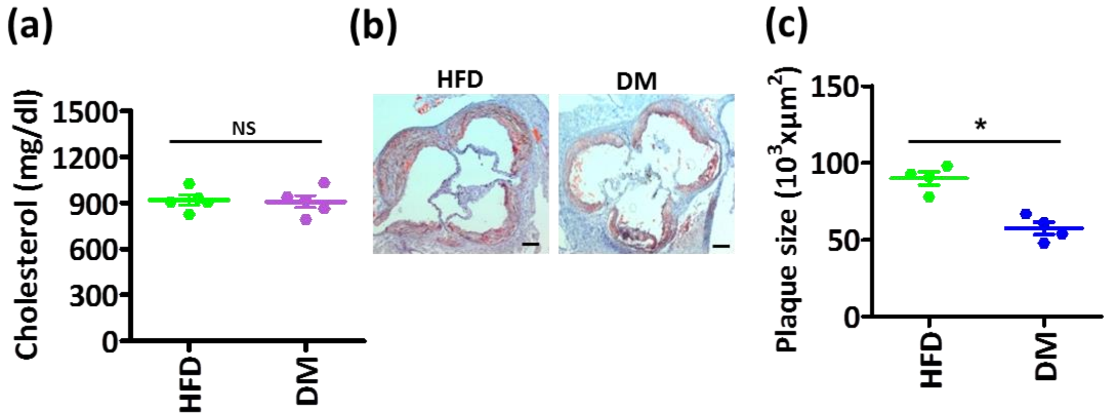

Supplementary Figure 2. Subgroup analyses of plaque size in hyperlipidaemic versus hyperglycaemic ApoE-/- mice matched for total plasma cholesterol levels (corresponding to Figure 1).

Total plasma cholesterol levels in selected ApoE-/- HFD (high fat diet, no streptozotocin) and ApoE-/- DM mice (normal chow diet, streptozotocin injections) matched for total plasma cholesterol levels (**a**). Representative Oil red O staining images of aortic root lesion (**b**) and dot-plot reflecting plaque size (**c**) in these selected mice.

Data shown as dot-plots represent mean ± SEM of 4 mice per group; NS: not significant, **P<0.01; a, c: unpaired t-test.

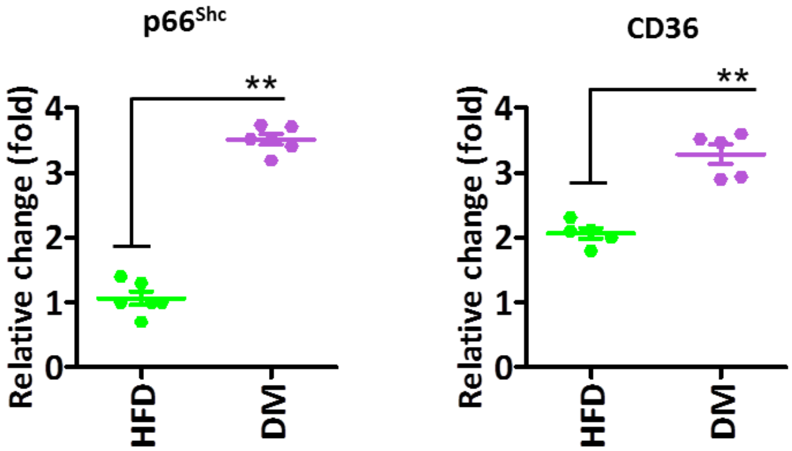

Supplementary Figure 3. qRT-PCR determination of p66^{Shc} and CD36-expression in plaque-associated macrophages (corresponding to Figure 2).

p66^{Shc} and CD36-expression are increased in laser micro dissected plaque-associated macrophages of ApoE-/- DM mice as compared to plaque-associated macrophages of ApoE-/- HFD mice. Dot-plot summarizing data (mean ± SEM) of quantitative real time-PCR showing relative fold change expression for p66^{Shc} (left) and CD36 (right), β-actin was used for normalisation; **P<0.01; unpaired t-test.

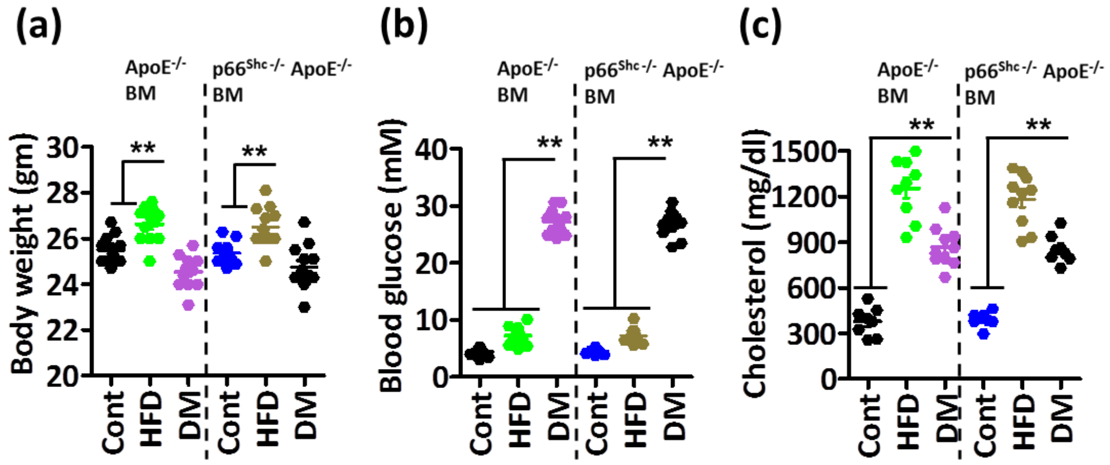

Supplementary Figure 4. Body weight and blood glucose levels following bone-marrow transplantation (corresponding to Figure 4).

Transplantation of $p66^{Shc}$-/- ApoE-/- bone marrow into ApoE-/- mice had no impact on body weight (**a**), blood glucose levels (**b**), or serum cholesterol levels (**c**) in comparison to ApoE-/- mice transplanted with $p66^{Shc}$ wild-type ApoE-/- bone marrow (ApoE-/- BM). Genotype of the transplanted bone marrow is shown on top.

Cont: normoglycaemic ApoE-/- mice with normal chow diet; HFD: ApoE-/-mice with high fat diet; DM: hyperglycaemic ApoE-/- mice. Data shown as dot-plots represent mean ± SEM of 6-10 mice per group; **P<0.01; two-way ANOVA with Bonferroni adjusted post-hoc comparison of ApoE-/- (Cont, HFD, DM) versus $p66^{Shc-/-}$ ApoE-/- (Cont, HFD, DM, respectively) recipient mice.

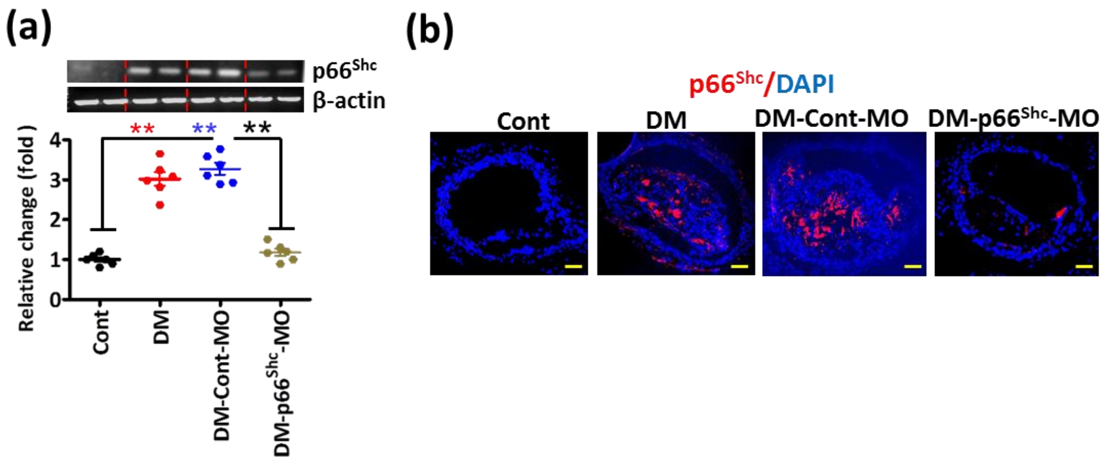

Supplementary Figure 5. *In vivo* knockdown of p66^{Shc} in diabetic ApoE-/- mice using vivo-morpholinos (corresponding to Figure 5).

In vivo knockdown of p66^{Shc} in diabetic ApoE-/- mice using vivo-morpholinos (p66^{Shc}-MO; mismatched control morpholino: Cont-MO) reduces p66^{Shc} expression in atherosclerotic plaques. Representative reverse-transcriptase-PCR images showing aortic expression of p66^{Shc} (**a**, top; β-actin: loading control) and dot-plot summarizing data (**a**, bottom). Representative co-immunofluorescence images for p66^{Shc} (red) and DAPI counterstain (blue) within brachiocephalic artery lesions.

Cont: normoglycaemic ApoE-/- mice with normal chow diet; DM: hyperglycaemic ApoE-/- mice; DM-Cont-MO: DM mice treated with mismatch control morpholinos; DM-p66^{Shc}-MO: ApoE-/- DM mice treated with p66^{Shc} vivo-morpholinos. Data shown as dot-plots represent mean ± SEM of 6 mice per group; **P<0.01; one-way ANOVA with Bonferroni adjusted post-hoc comparison of DM and DM-Cont-MO versus Cont and DM-p66^{Shc}-MO versus DM-Cont-MO. Uncropped reverse-transcriptase-PCR gel images for Supplementary Figure 5a are provided in Supplementary Figure 24.

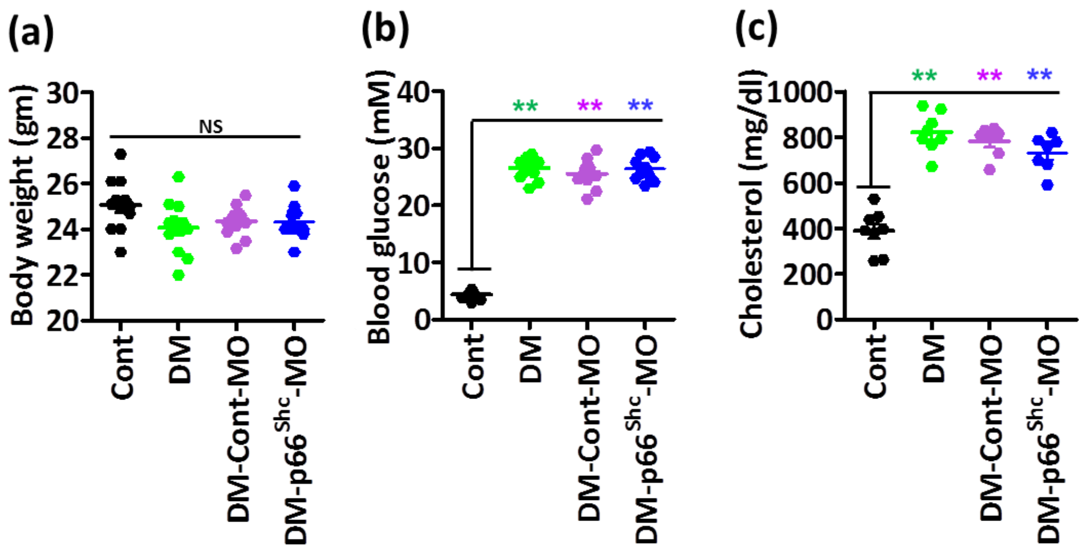

Supplementary Figure 6. Body weight and blood glucose levels following *in vivo* p66^{Shc} knockdown (corresponding to Figure 5).

In ApoE-/- DM mice treated with p66^{Shc} vivo-morpholino (DM-p66^{Shc}-MO) or mismatch control morpholino (DM-Cont-MO) body weight (**a**), blood glucose levels (**b**), and total plasma cholesterol levels (**c**) are comparable to untreated diabetic mice (DM). Non-diabetic control ApoE-/- mice (Cont) shown for comparison.

Cont: normoglycaemic ApoE-/- mice with normal chow diet; DM: hyperglycaemic ApoE-/- mice; DM-Cont-MO: DM mice treated with mismatch control morpholinos; DM-p66^{Shc}-MO: ApoE-/- DM mice treated with p66^{Shc} vivo-morpholinos. Data shown as dot-plots represent mean ± SEM of at least 8 mice per group; NS: not significant, **P<0.01; one-way ANOVA with Bonferroni adjusted post-hoc comparison of DM, DM-Cont-MO and DM-p66^{Shc}-MO versus Cont.

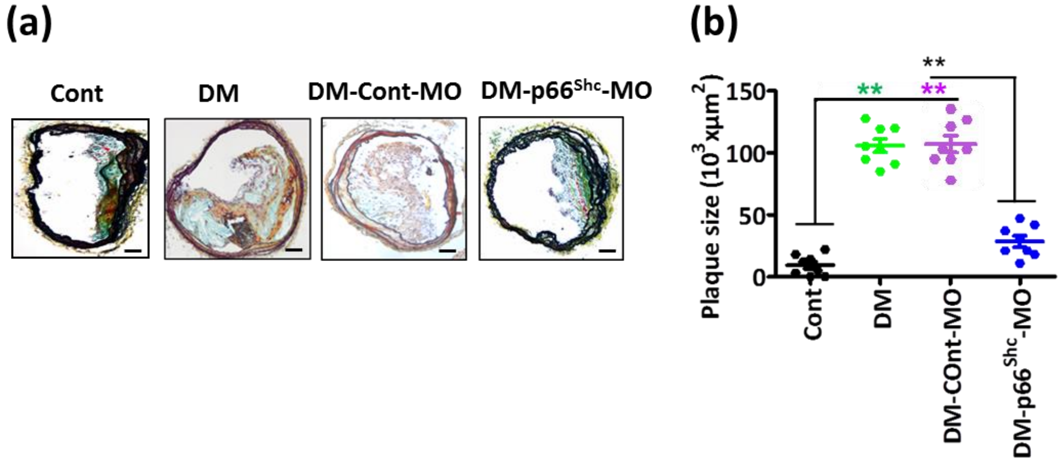

Supplementary Figure 7. A pivotal function of p66^{Shc} expression in hyperglycaemia-induced atherosclerosis (corresponding to Figure 5).

In vivo knockdown of p66^{Shc} using vivo-morpholinos (p66^{Shc}-MO; mismatched control morpholino: Cont-MO) induces atherosclerotic plaques regression in hyperglycaemic mice. Representative images of MOVAT stained brachiocephalic artery (**a**) and dot-plot summarizing data (**b**).

Cont: normoglycaemic ApoE-/- mice with normal chow diet; DM: hyperglycaemic ApoE-/- mice. DM-Cont-MO: ApoE-/- DM mice treated with mismatch control morpholinos; DM-p66^{Shc}-MO: ApoE-/- DM mice treated with p66^{Shc} vivo-morpholinos; mean ± SEM of 8 mice per group; **P<0.01; one-way ANOVA with Bonferroni adjusted post-hoc comparison of DM and DM-Cont-MO versus Cont and DM-p66^{Shc}-MO versus DM-Cont-MO.

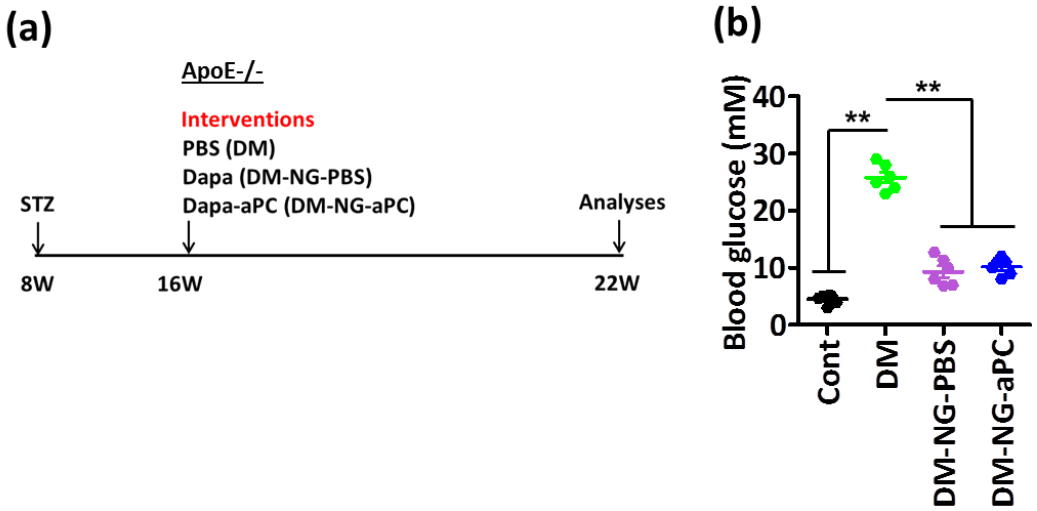

Supplementary Figure 8. Effect of dapagliflozin and aPC treatment on blood glucose levels in ApoE-/- DM mice (corresponding to Figure 6).

a. Experimental scheme.

b. SGLT2 inhibitor treatment (dapagliflozin) for 6 weeks after 16 weeks of persistent hyperglycaemia (DM-NG-PBS) markedly reduces blood glucose levels. Additional treatment with aPC (DM-NG-aPC) has no further effect on blood glucose levels.

Cont: normoglycaemic ApoE-/- mice with normal chow diet; DM: hyperglycaemic ApoE-/- mice; DM-NG-PBS: SGLT2 inhibitor and PBS treated ApoE-/- DM mice; DM-NG-aPC: DM-NG mice with concomitant aPC and SGLT2 inhibitor treatment. Data shown as dot-plots represent mean ± SEM of 6-8 mice per group (b); **P<0.01; b, one-way ANOVA with Bonferroni adjusted post-hoc comparison of DM versus Cont and DM-NG-aPC and DM-NG-PBS versus DM mice.

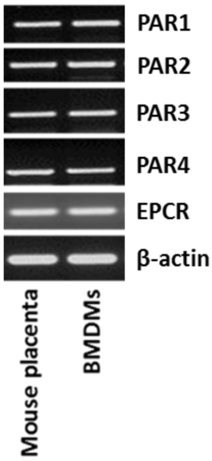

Supplementary Figure 9. BMDMs express protease activated receptors (PARs) and endothelial protein C receptor (EPCR) (corresponding to Figure 6).

Bone marrow derived macrophages (BMDMs) readily express all PARs (PAR1-4) and EPCR; reverse-transcriptase-PCR gel images representative of three independent experiments, mouse placenta tissue is used as a positive control (β-actin: loading control).

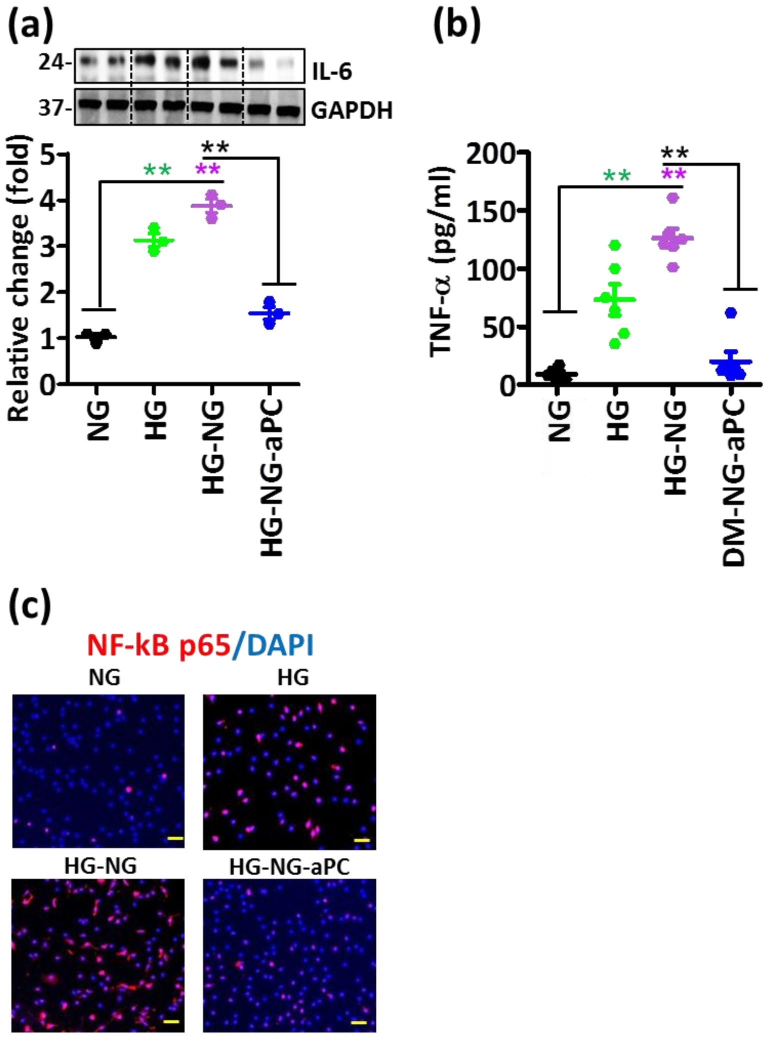

Supplementary Figure 10. Activated protein C reverses hyperglycaemia induced sustained IL-6, TNF-α and NF-κB p65 protein levels in BMDMs (corresponding to Figure 7).

Representative immunoblot images of IL-6 (**a**, out of 3 independent repeat experiments each with two technical replicates, top; GAPDH: loading control) and dot-plot summarizing data representing of three independent experiments (**a**, bottom). Dot-plot summarizing data of TNF-α level determined by ELISA representing six independent experiments (**b**). Representative co-immunofluorescence images (**c**, out of 3 independent repeat experiments) for NF-κB p65 (red) and DAPI counterstain (blue).

NG: normal glucose (5 mM glucose plus 20 mM mannitol, 48 hr), HG: high glucose (25 mM, 48 hr), HG-NG: HG (48 hr) followed by NG (24 hr) condition; HG-NG-aPC: HG-NG conditions with additional exposure to aPC (20 nM) during the last 24 hr). **P<0.01; a,b, one-way ANOVA with Bonferroni adjusted post-hoc comparison of HG and HG-NG versus NG and HG-NG-aPC versus HG-NG. Uncropped immunoblot images for Supplementary Figure 10a are provided in Supplementary Figure 24.

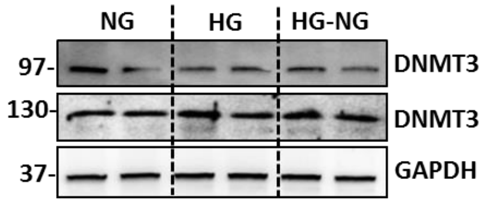

Supplementary Figure 11. Expression of DNMT3A and DNMT3B in BMDMs (corresponding to Figure 8).

In glucose (25 mM) stressed BMDMs protein expression of DNA methyltransferases DNMT3A and DNMT3B remains normal. Representative immunoblot images (out of 4 independent repeat experiments with two technical replicates each); GAPDH was used as loading control. ncropped immunoblot images are provided in Supplementary Figure 24.

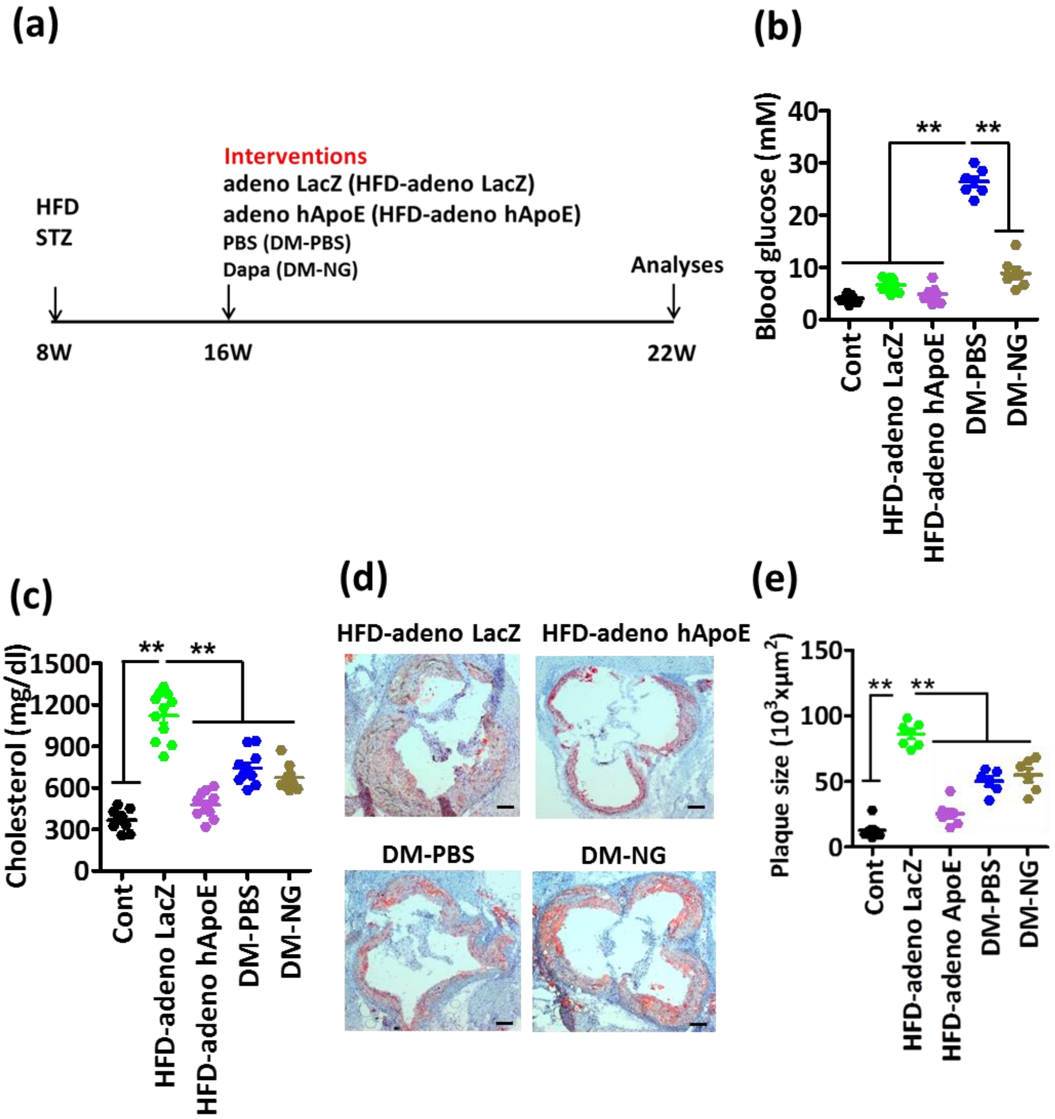

Supplementary Figure 12. Atherosclerotic plaque regression is impaired in diabetic, but not hyperlipidaemic mice (corresponding to Figure 9).

a. Experimental scheme.

b,c. Dot-plot summarizing blood glucose (b) and total plasma cholesterol levels in experimental mice.

d,e. Plaque size regressed in HFD-adeno^{hApoE} versus HFD-adeno^{LacZ} mice but remained stable in DM-NG versus DM-PBS mice. Representative Oil Red O staining of aortic roots lesion (**d**) and dot-plot summarizing results (**e**).

Cont: normoglycaemic ApoE-/- mice with normal chow diet; HFD-adeno LacZ: HFD ApoE-/- mice treated with control LacZ adenovirus; HFD-adeno hApoE: HFD ApoE-/- mice treated with human ApoE expressing adenovirus; DM-NG: ApoE -/- DM mice treated with SGLT2 inhibitor weeks 16 to 22; DM-PBS: DM mice treated with PBS. Data shown represent mean ± SEM of 6-10 mice per group (b, c, e); $^{**}P<0.01$; one-way ANOVA with Bonferroni adjusted post-hoc comparison of DM-PBS versus Cont, HFD-adeno LacZ, HFD-adeno ApoE, and DM-NG (a), HFD-adeno LacZ versus Cont, DM-PBS, DM-NG, HFD-adeno ApoE (c,e).

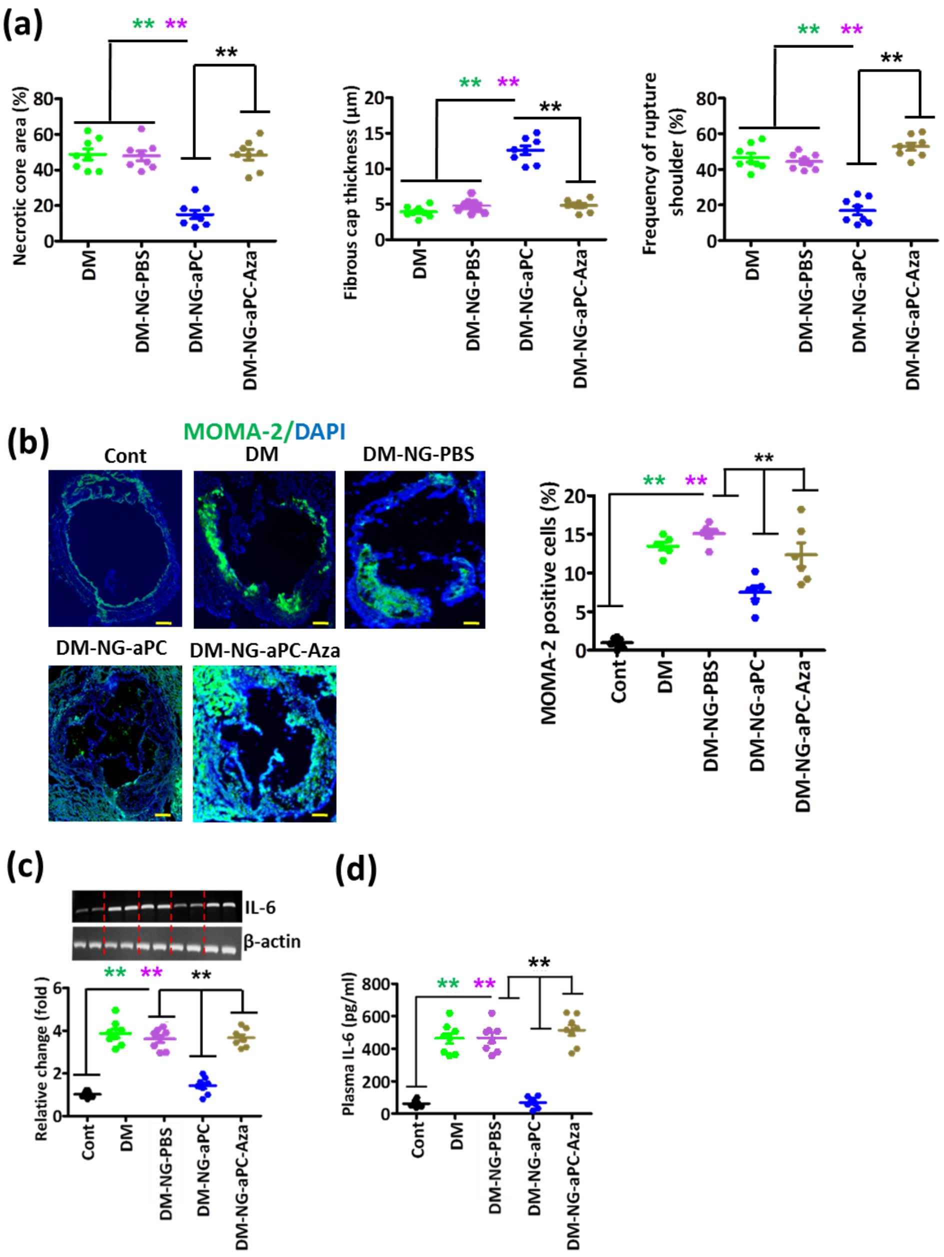

Supplementary Figure 13. aPC reverses hyperglycaemia-induced plaque instability and persistent IL-6 expression (corresponding to Figure 9).

a. Morphometric analyses of haematoxylin and eosin stained images showing necrotic core area (left panel), fibrous cap thickness (middle panel), and frequency of ruptured shoulders (right panel).

b. Representative images showing immunofluorescence staining of macrophages (MOMA-2, green; DAPI nuclear counterstain, blue) within lesions (left panel) and dot-plot summarizing data (right panel).

c, d. Representative reverse-transcriptase-PCR gel images (c, top, β-actin as loading control) and dot-plot summarizing for IL-6 protein expression (c). Dot-plot summarizing data of plasma IL-6 levels (d). Cont: normoglycaemic ApoE-/- mice with normal chow diet; DM: hyperglycaemic ApoE-/- mice; DM-NG-PBS: SGLT2 inhibitor and PBS treated ApoE-/- DM mice; DM-NG-aPC: SGLT2 inhibitor and aPC treated ApoE-/- DM mice; DM-NG-aPC-Aza: SGLT2 inhibitor, aPC and 5-azacytidine treated ApoE-/- DM mice. Data shown represent mean ± SEM of 8-10 mice per group; **P<0.01(one-way ANOVA with Bonferroni adjusted post-hoc comparison of DM-NG-aPC versus DM and DM-NG-PBS and DM-NG-aPC-Aza versus DM-NG-aPC (a), DM-NG-PBS and DM versus Cont, DM-

NG-aPC versus DM-NG-PBS and DM-NG-aPC-Aza (b-d). Uncropped reverse-transcriptase-PCR gel images for Supplementary Figure 13c are provided in Supplementary Figure 25.

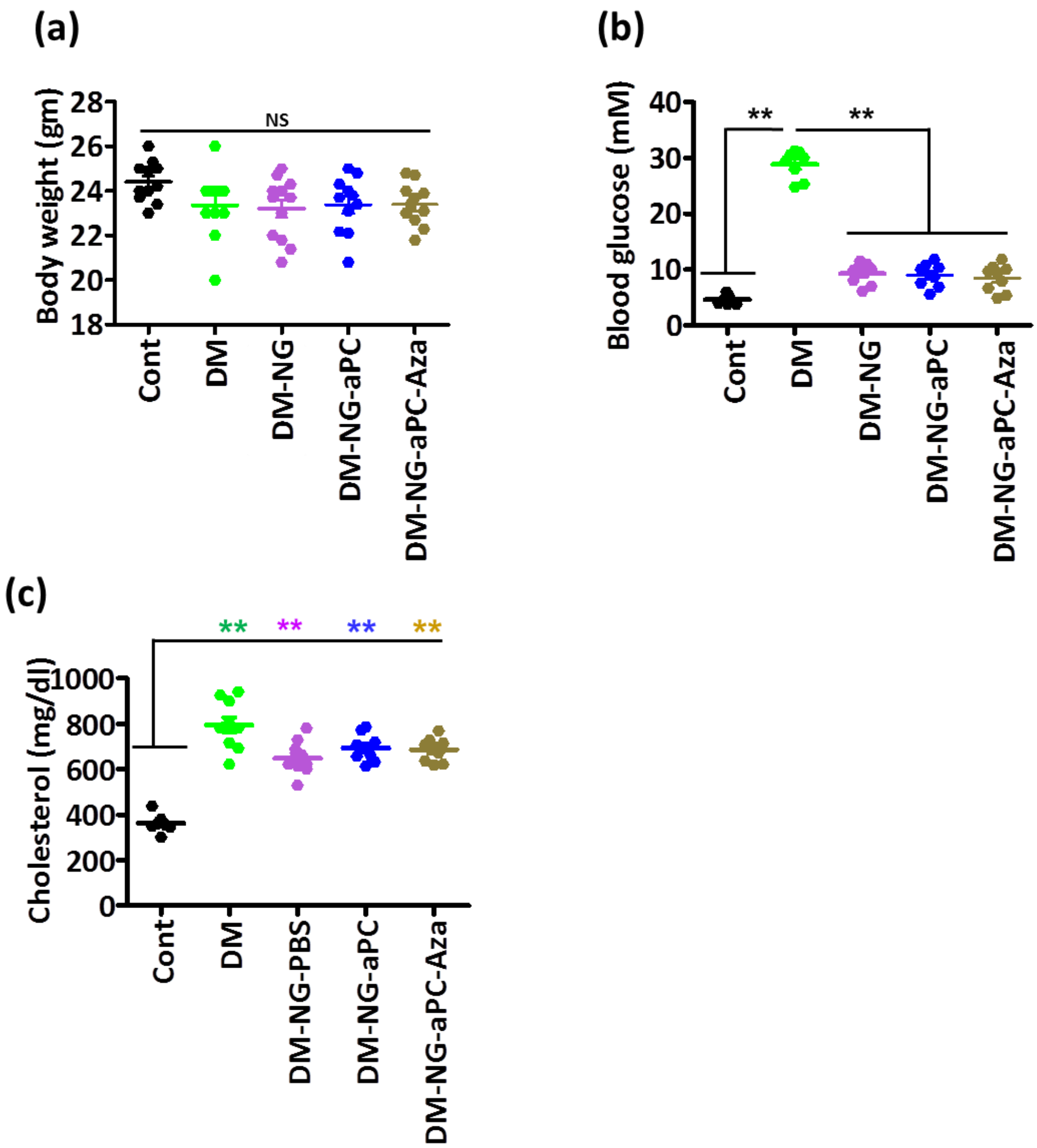

Supplementary Figure 14. Effect of 5-azacytidine on body weight, blood glucose, and total plasma cholesterol levels in ApoE-/- DM mice (corresponding to Figure 9).

Treatment of mice with 5-azacytidine (Aza) in addition to aPC and blood glucose normalization (DM-NG-aPC-Aza) has no impact on body weight (**a**, compared to all other groups), blood glucose levels (**b**, compared to DM-NG-PBS and DM-NG-aPC) or total plasma cholesterol levels (**c**, compared to DM, DM-NG-PBS and DM-NG-aPC).

Cont: normoglycaemic ApoE-/- mice with normal chow diet; DM: hyperglycaemic ApoE-/- mice; DM-NG-PBS: SGLT2 inhibitor and PBS treated ApoE-/- DM mice; DM-NG-aPC: SGLT2 inhibitor and aPC treated ApoE-/- DM mice; DM-NG-aPC-Aza: SGLT2 inhibitor, aPC and 5-azacytidine treated ApoE-/- DM mice. Data shown as dot-plots represent mean ± SEM of 8-10 mice per group; NS: not significant, **P<0.01; one-way ANOVA with Bonferroni adjusted post-hoc comparison of DM, DM-NG-PBS, DM-NG-aPC and DM-NG-aPC-Aza versus Cont (a,c), DM versus Cont, DM-NG-PBS, DM-NG-aPC and DM-NG-aPC-Aza (b).

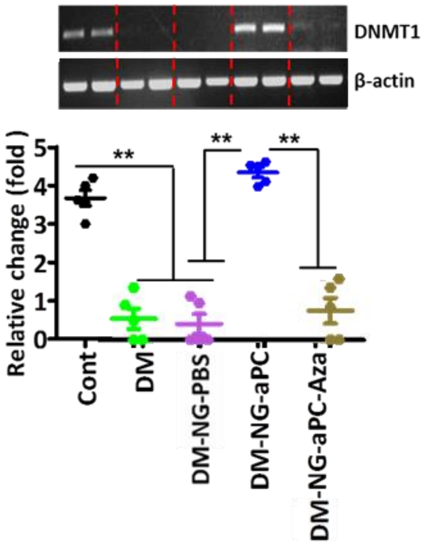

Supplementary Figure 15. DNMT1 mRNA expression in aorta (corresponding to Figure 9). Representative semiquantitative reverse-transcriptase-PCR gel image of DNMT1; β-actin as loading. Cont: normoglycaemic ApoE-/- mice with normal chow diet; DM: hyperglycaemic ApoE-/- mice; DM-NG-PBS: SGLT2 inhibitor and PBS treated ApoE-/- DM mice; DM-NG-aPC: SGLT2 inhibitor and aPC treated ApoE-/- DM mice; DM-NG-aPC-Aza: SGLT2 inhibitor, aPC and 5-azacytidine treated ApoE-/- DM mice. Data shown as dot-plots represent mean ± SEM of 6 mice per group; **P<0.01; one-way ANOVA with Bonferroni adjusted post-hoc comparison of DM and DM-NG-PBS versus Cont and DM-NG-aPC versus DM-NG-aPC-Aza and DM-NG-PBS. Uncropped reverse-transcriptase-PCR gel images are provided in Supplementary Figure 25.

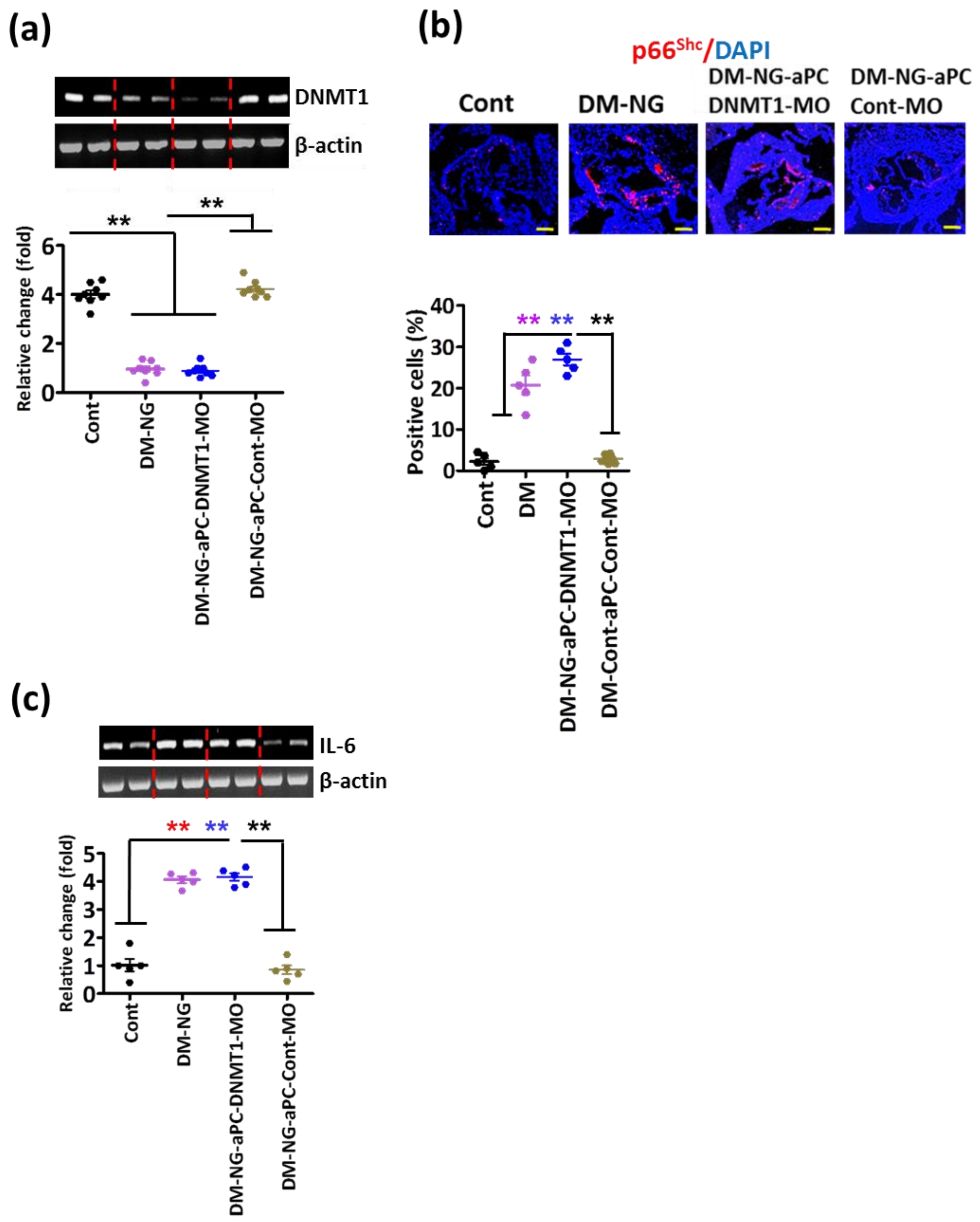

Supplementary Figure 16. Effects of aPC treatment in mice following DNMT1 *in vivo* silencing (corresponding to Figure 10).

a. Representative reverse-transcriptase-PCR gel images for DNMT1 expression (**a**, β-actin as loading control, top) and dot-plot summarizing data (**a**, bottom).

b. Representative co-immunofluorescence images (top panel) for p66^{Shc} (red), DAPI nuclear counterstain (blue), and dot-plot summarizing data (bottom panel).

c. Representative reverse-transcriptase-PCR gel images for IL-6 expression (β-actin as loading control, top) and dot-plot summarizing data (bottom).

Cont: normoglycaemic ApoE-/- mice with normal chow diet; DM: hyperglycaemic ApoE-/- mice; DM-NG: SGLT2 inhibitor treated ApoE-/- DM mice; DM-NG-aPC-DNMT1-MO: DM-NG mice with concomitant SGLT2 inhibitor, aPC, and DNMT1-MO treatment; DM-NG-aPC-Cont-MO: DM-NG mice with concomitant SGLT2 inhibitor, aPC, and control morpholino treatment. Data shown represent mean ± SEM of 8-10 mice per group; **P<0.01; one-way ANOVA with Bonferroni adjusted post-hoc comparison of DM-NG and DM-NG-aPC-DNMT1-MO versus Cont and DM-NG-aPC-DNMT1-MO versus DM-NG-aPC-Cont-MO). Uncropped reverse-transcriptase-PCR gel images for Supplementary Figure 16a,c are provided in Supplementary Figure 26.

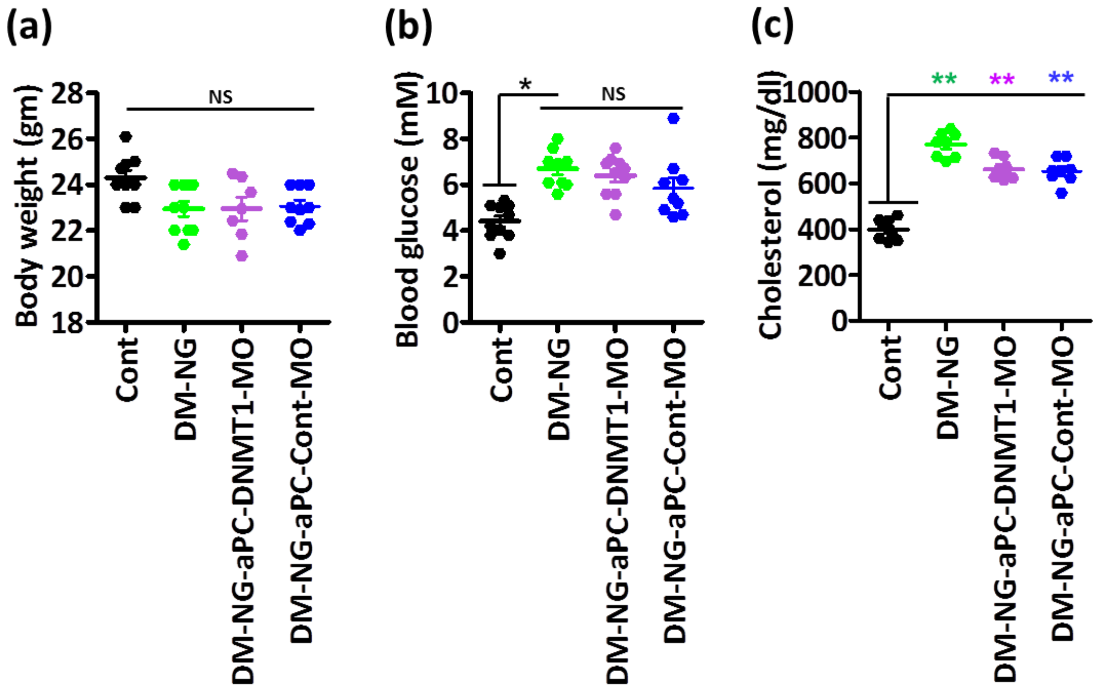

Supplementary Figure 17. Body weight, blood glucose, and blood glucose levels following *in vivo* knockdown of DNMT1 (corresponding to Figure 10).

DNMT1 vivo morpholino and control morpholino have no significant effects on body weight (**a**), blood glucose (**b**), and total plasma cholesterol levels (**c**) when compared to DM-NG mice.

Cont: normoglycaemic ApoE-/- mice with normal chow diet; DM: hyperglycaemic ApoE-/- mice; DM-NG: SGLT2 inhibitor treated ApoE-/- DM mice; DM-NG-aPC-DNMT1-MO: DM-NG mice with concomitant SGLT2 inhibitor, aPC, and DNMT1-MO treatment; DM-NG-aPC-Cont-MO: DM-NG mice with concomitant SGLT2 inhibitor, aPC, and control morpholino treatment. Data shown as dot-plots represent mean ± SEM of 8-10 mice per group; NS: not significant, *P<0.05, **P<0.01; one-way ANOVA with Bonferroni adjusted post-hoc comparison of Cont versus DM-NG, DM-NG-aPC-DNMT1-MO, and DM-NG-aPC-Cont-MO (a,c) and DM-NG versus Cont and DM-NG-aPC-DNMT1-MO and DM-NG-aPC-Cont-MO versus DM-NG-PBS (b).

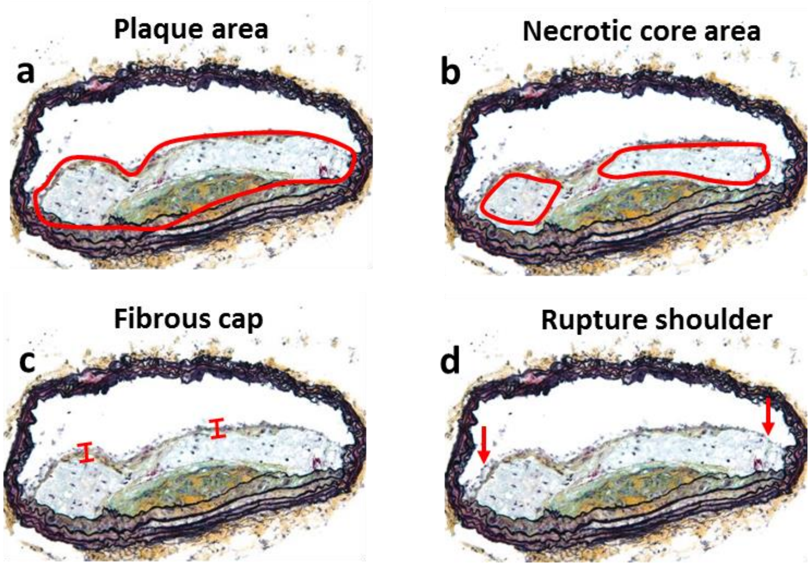

Supplementary Figure 18. Morphometric analysis of MOVAT stained atherosclerotic plaque morphology.

Representative images showing MOVATs staining of brachiocephalic arteries. Morphometric analyses of MOVATs reflecting plaque area (a), necrotic core area (b), fibrous caps thickness (c), and ruptured shoulders (d); size bars: 20 µm.

for Figure 2a

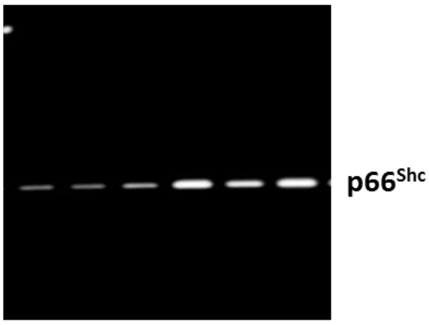

for Figure 2a

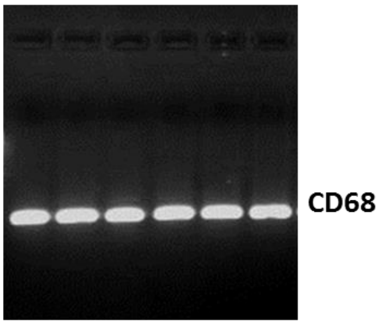

for Figure 2a

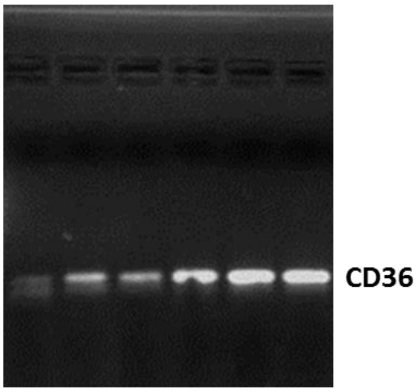

for Figure 2a

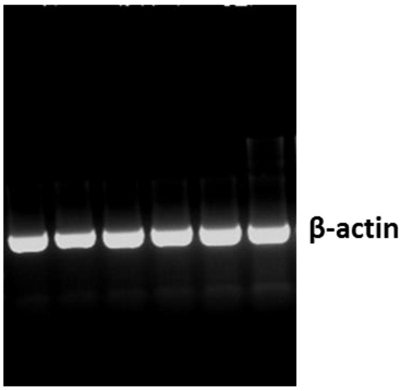

for Figure 2c

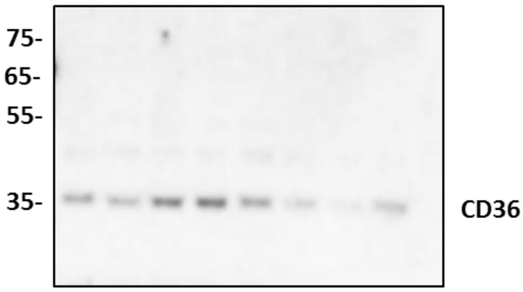

for Figure 2c

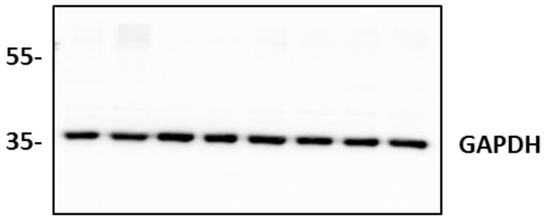

Supplementary Figure 19. Full-sized reverse-transcriptase-PCR gel images and immunoblots corresponding to Figure 2a and Figure 2c, respectively.

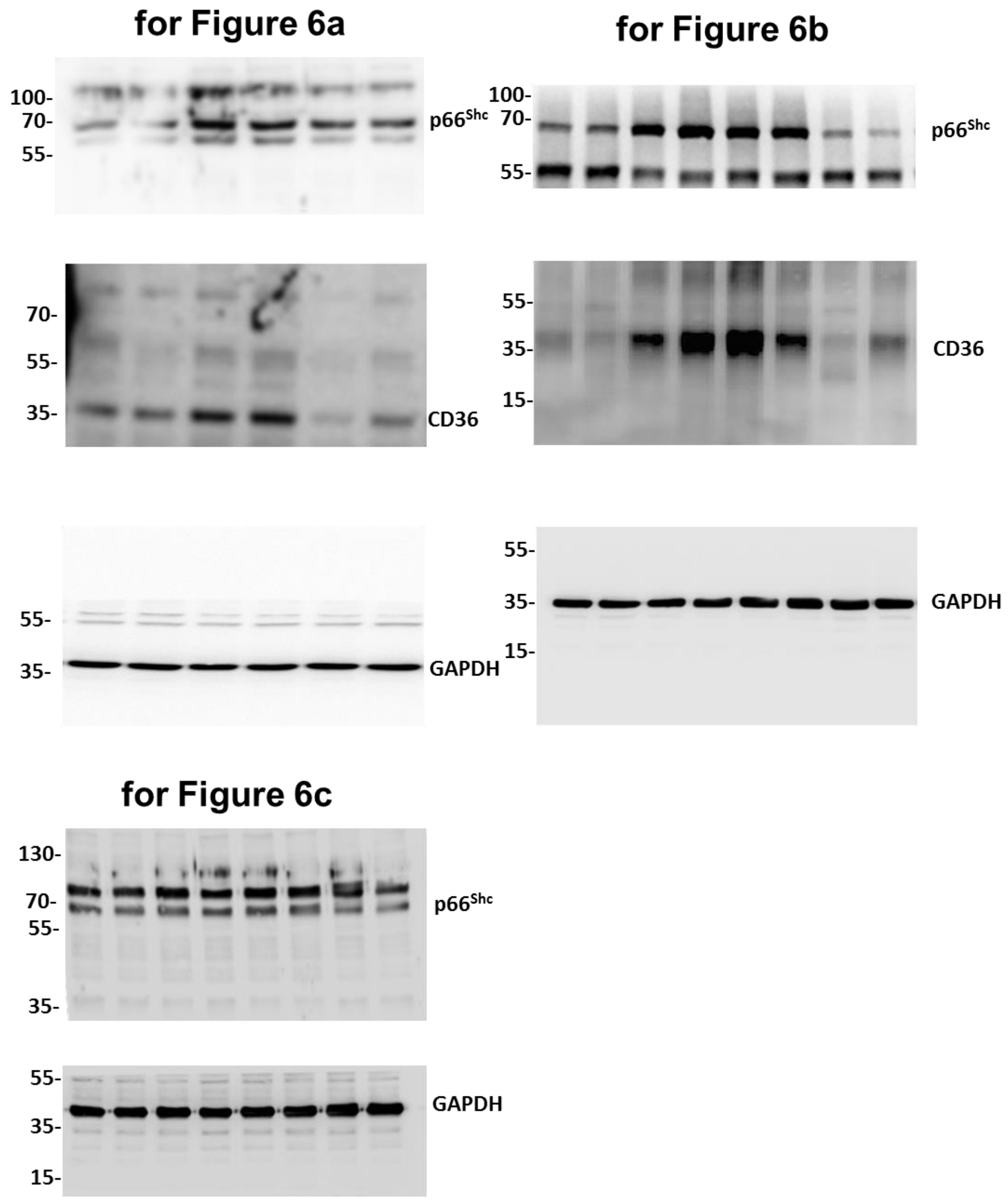

Supplementary Figure 20. Full-sized immunoblots corresponding to Figure 6a-c.

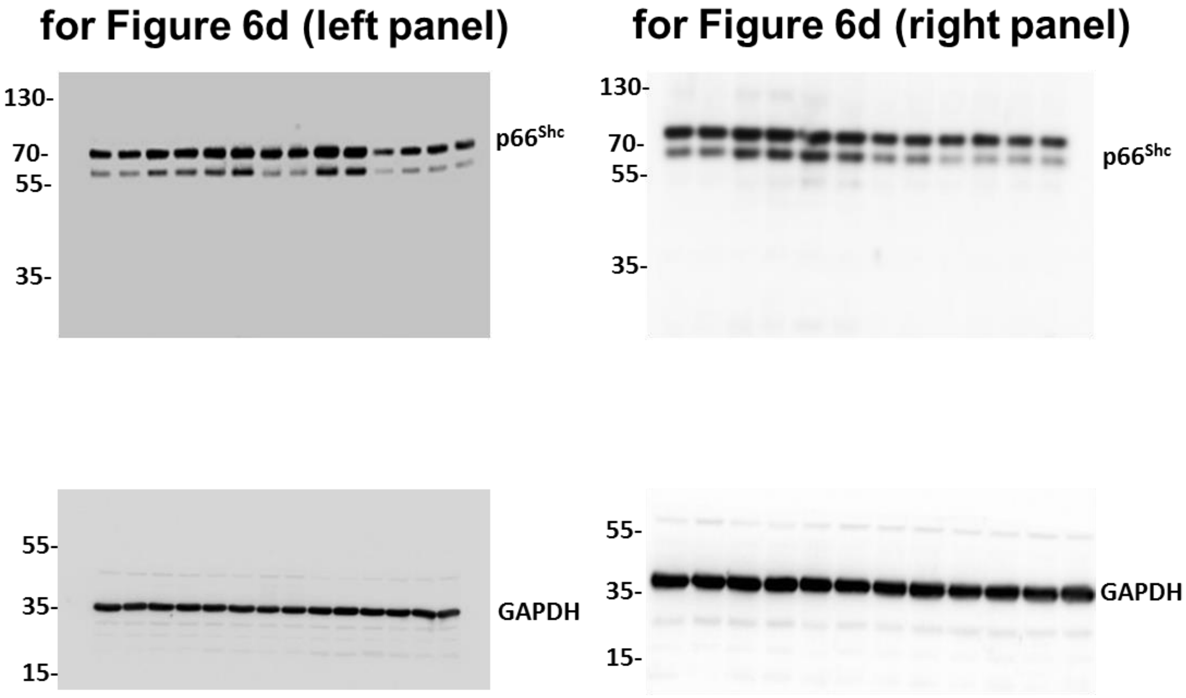

Supplementary Figure 21. Full-sized immunoblots corresponding to Figure 6d.

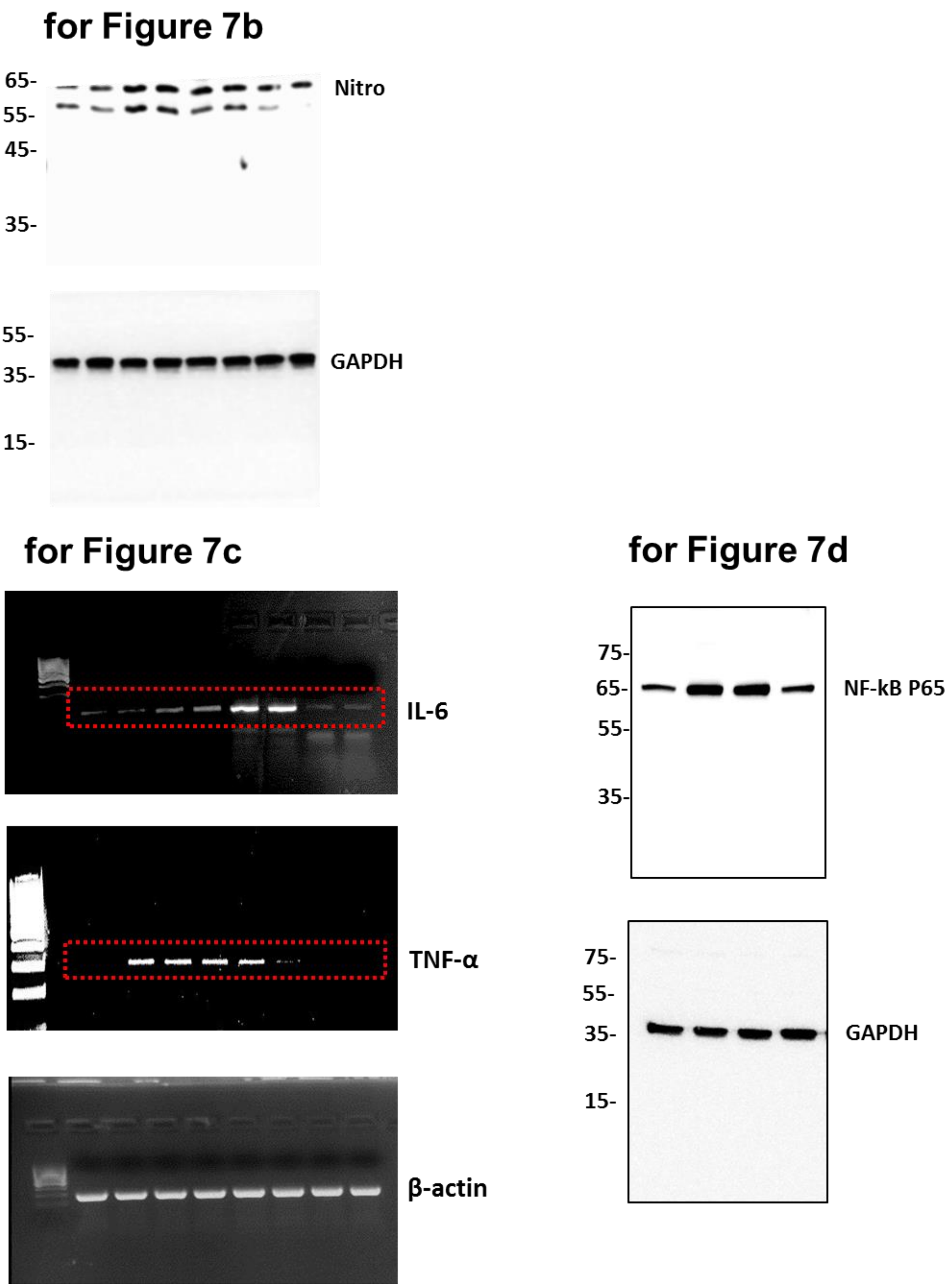

Supplementary Figure 22. Full-sized immunoblots corresponding to Figure 7b,d and reverse-transcriptase-PCR gel images corresponding to Figure 7c.

for Figure 8a

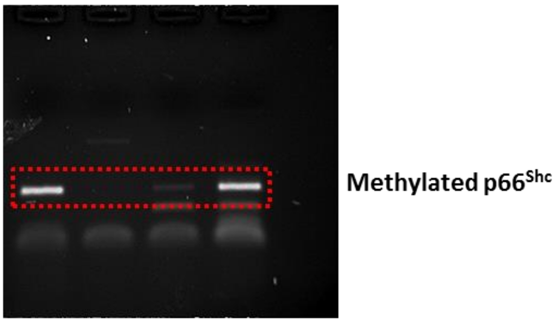

for Figure 8b

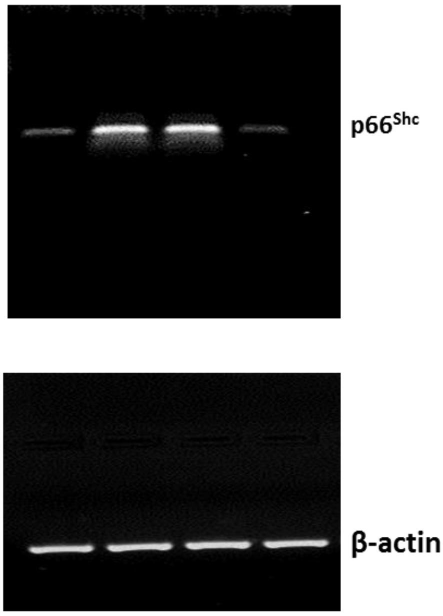

for Figure 8c

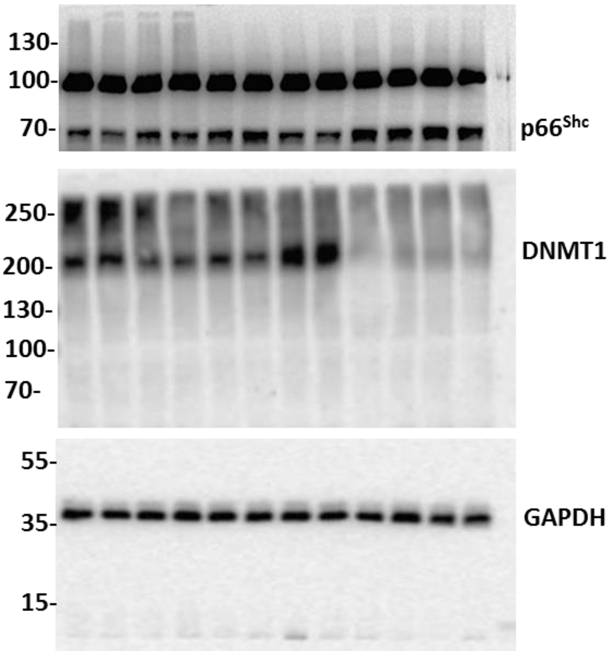

Supplementary Figure 23. Full-sized reverse-transcriptase-PCR gel images corresponding to Figure 8a, b and immunoblots corresponding to Figure 8c.

for Supplementary Figure 5

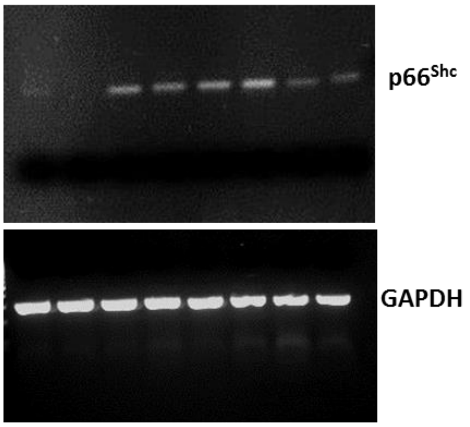

for Supplementary Figure 10

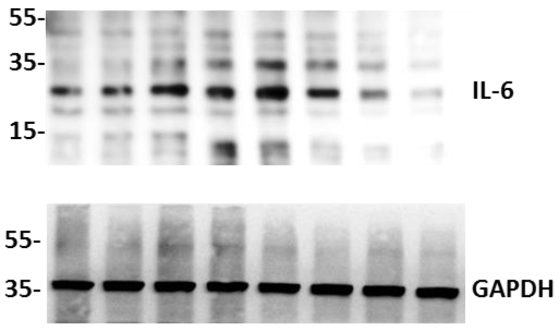

for Supplementary Figure 11

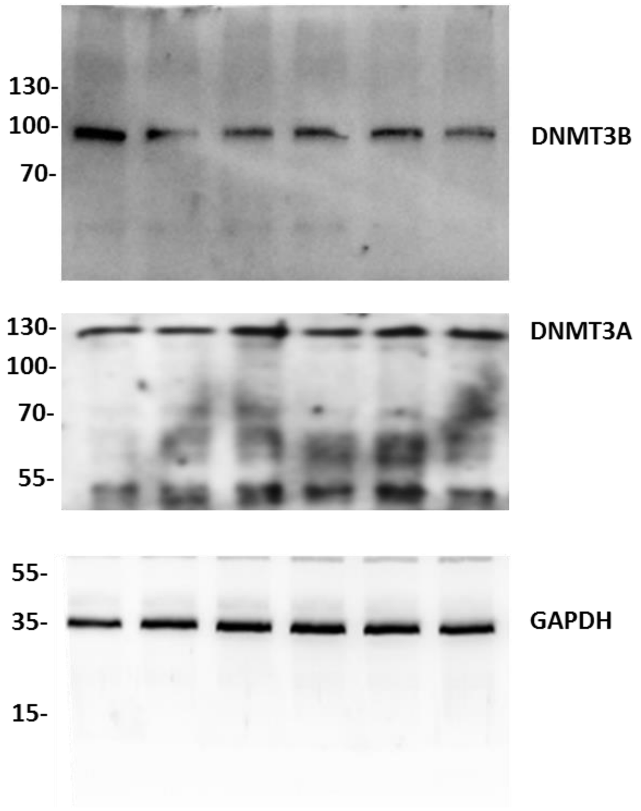

Supplementary Figure 24. Full-sized reverse-transcriptase-PCR gel images corresponding to Figure 7b-d and immunoblots corresponding to supplementary Figure 5, supplementary Figure 10, and supplementary Figure 11.

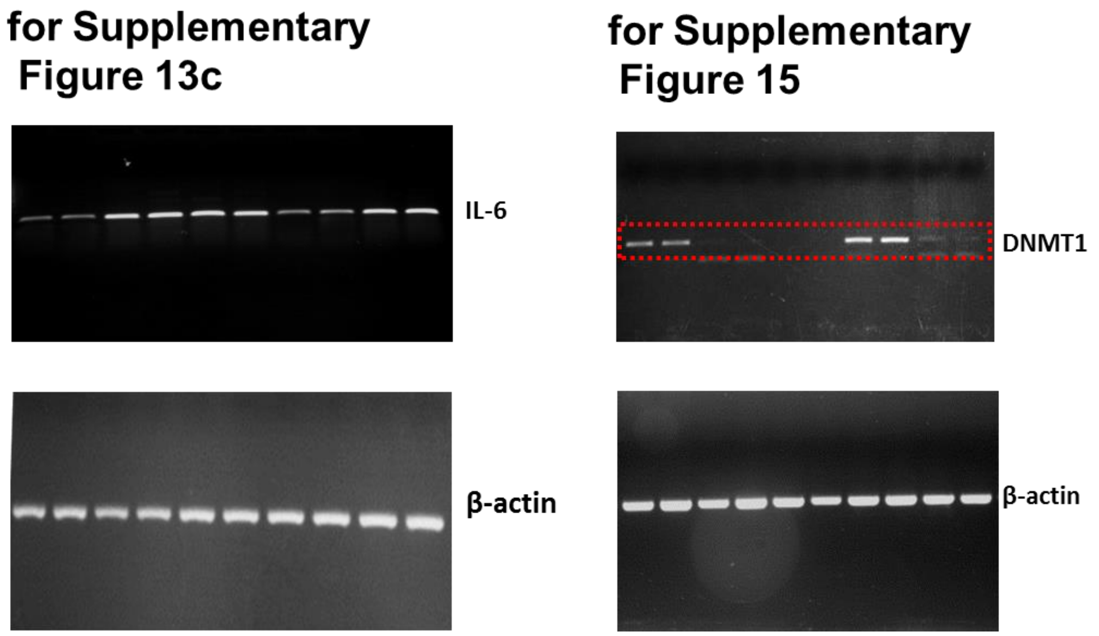

Supplementary Figure 25. Full-sized reverse-transcriptase-PCR gel images corresponding to supplementary Figure 13c and supplementary Figure 15.

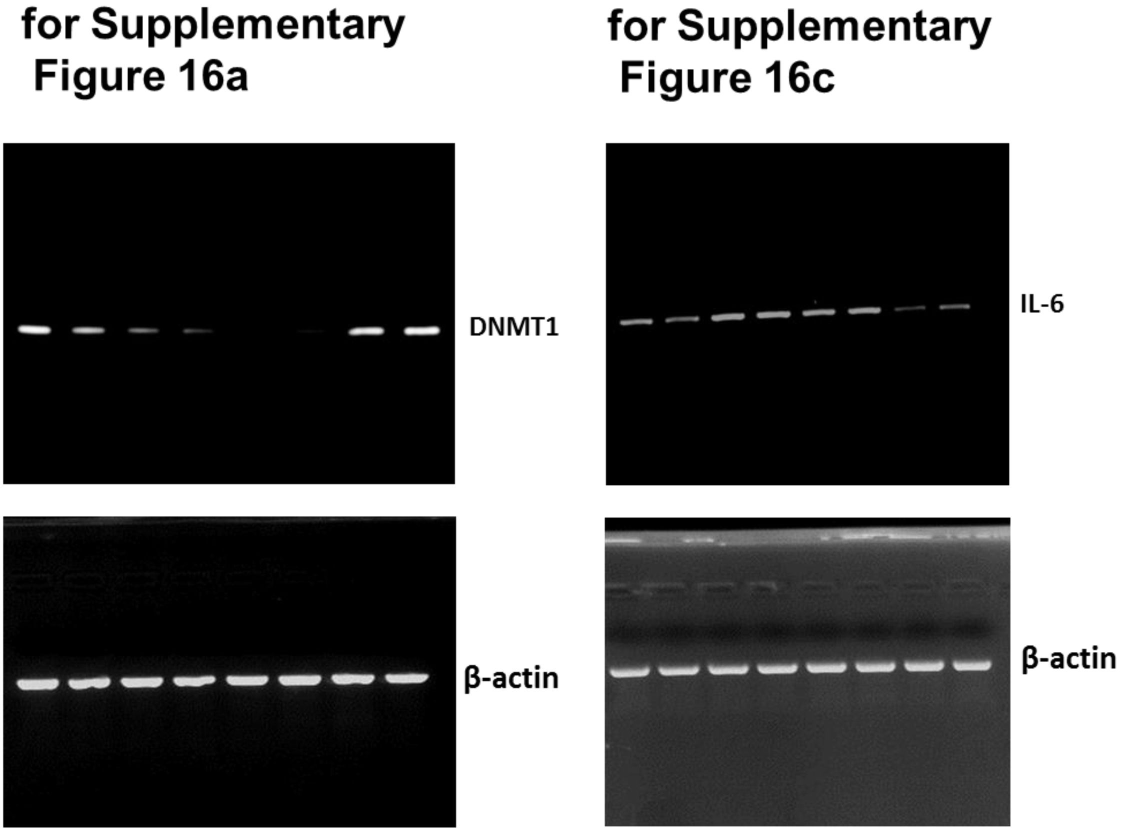

Supplementary Figure 26. Full-sized reverse-transcriptase-PCR gel images corresponding to supplementary Figure 16 a,c.

**Target**	**Primer Sequence**
**methylated ($p66^{Shc}$)**	5’TTTTCGTTTTTTGGGTTC3’<br>5’TACGTATTCCTACCGAACG3’
**unmethylated ($p66^{Shc}$)**	5’TAATTTTTGTTTTTTGGGTTT3’<br>5’TACATATTCCTACCAAACACAA3’
**mPAR1**	5'CCAGCCAGAATCAGAGAGGA3'<br>5'TCGGAGATGAAGGGAGGAG3'
**mPAR2**	5'CCAGGAAGAAGGCAAACATC3'<br>5’TGTCCCCCACCAATACCTC3'
**mPAR3**	5'CATCCTGCTGTT TGTGGTTG3'<br>5'TACCCAGTTGTT GCCATTGA3'
**mPAR4**	5'GCAGACCTTCCGATTAGCTG3'<br>5'CACTGCCGAGAACAGTACCA3'
**DNMT3b**	5'TTCAGTGACCAGTCCTCAGACACGAA3'<br>5'TCAGAAGGCTGGAGACCTCCCTCTT3'
**β-actin**	5'CTAGACTTCGAGCAGGAGATGG3'<br>5'GCTAGGAGCCAGAGCAGTAATC3'
**TNF-alpha**	5'ACAGAAAGCATGATCCGCGA3'<br>5'TCCACTTGGTGGTTTGCTACG3'
**IL6**	5'GCCTTCTTGGGACTGATGCT3'<br>5'TGCCATTGCACAACTCTTTTC3'
**MCP1**	5'GCTGTAGTTTTTGTCACCAAGC3'<br>5'AAGGCATCACAGTCCGAGTC3'
**CD36**	5'TGAATGGTTGAGACCCCGTG3'<br>5'CGTGGCCCGGTTCTACTAAT3'
**$p66^{Shc}$**	5'ACTACCCTGTGTTCCTTCTTTC3'<br>5'TCGGTGGATTCCTGAGATACTGT3'

Supplementary Table 1. List of primers used in the current study.

Parameter	Group statistics		*P*-value
	**Non-DM** (N=10)	**DM** (N=10)	Non-DM vs DM
**Age (years)**	63.39 ± 1.214	62.17 ± 1.341	ns
**Sex (M/F)**	8/2	7/3	ns
**Diabetes duration (years)**	-	15.33 ± 1.142	-
**HbA1c (%)**	4.88 ± 0.053	7.65 ± 0.234	<0.01
**CAD (Y/N)**	5/5	6/4	ns
**RAS (Y/N)**	3/7	4/6	ns
**HTN (Y/N)**	5/5	4/6	ns
**Chol (mmol/L)**	6.51 ± 0.012	3.56 ± 0.190	0.02
**LDL (mmol/L)**	4.90 ± 0.571	2.41 ± 0.176	<0.01
**BMI (kg/m^2)**	32.91 ± 1.096	30.88 ± 0.7462	ns
**Smoking (Y/N)**	3/7	4/6	ns
**Oral antidiabetic medication (Y/N)**	-	8/2	-
**Insulin treatment (Y/N)**	-	6/4	-
**Lipid lowering drug (Y/N)**	6/4	2/8	<0.01
**antihypertensive drug (Y/N)**	5/5	4/6	ns
**Platelet activation inhibitors (Y/N)**	4/6	3/7	ns

Supplementary Table 2. Clinical characteristics of patients from which plaque biopsies were obtained. Data were obtained at the time of biopsy. Abbreviations: M: Male, F: Female, BMI: body mass index, Y: Yes, N: No, ACI: internal carotid artery, HTN: Hypertension, CAD: Coronary Artery Disease, RAS: Renal Artery Stenosis, Chol: Cholesterol. For statistical analysis of Sex the Fisher's exact test was used and data are shown as mean ± SEM. For all other parameters analyses were performed using the unpaired two-tailed student's *t*-test. For significant differences the *P*-values are shown (ns: not significant).
